# Replenishing co‐downregulated miR‐100‐5p and miR‐125b‐5p in malignant germ cell tumors causes growth inhibition through cell cycle disruption

**DOI:** 10.1002/1878-0261.13757

**Published:** 2024-11-10

**Authors:** Marta Ferraresso, Shivani Bailey, Luz Alonso‐Crisostomo, Dawn Ward, Christos Panayi, Zachary G. L. Scurlock, Harpreet K. Saini, Stephen P. Smith, James C. Nicholson, Anton J. Enright, Cinzia G. Scarpini, Nicholas Coleman, Matthew J. Murray

**Affiliations:** ^1^ Department of Pathology University of Cambridge UK; ^2^ EMBL‐European Bioinformatics Institute (EMBL‐EBI) Cambridge UK; ^3^ Department of Paediatrics University of Cambridge, Cambridge University Hospitals NHS Foundation Trust UK; ^4^ Department of Paediatric Haematology and Oncology Cambridge University Hospitals NHS Foundation Trust UK; ^5^ Department of Histopathology Cambridge University Hospitals NHS Foundation Trust UK

**Keywords:** germ cell tumor, *in vitro* models, methylation, microRNA, mRNA, testis

## Abstract

MicroRNAs (miRNAs) are short, nonprotein‐coding RNAs, and their expression is dysregulated in malignant germ cell tumors (GCTs). Here, we investigated the causes and consequences of downregulated miR‐99a‐5p/miR‐100‐5p (functionally identical) and miR‐125b‐5p levels in malignant GCTs regardless of age, site, or subtype. Quantitative RT‐PCR was used to assess miR‐99a‐5p/miR‐100‐5p, miR‐125b‐5p, and associated gene expression in malignant GCT tissues/cell lines [seminoma (Sem), yolk sac tumor (YST), embryonal carcinoma (EC)]. Cells were treated with demethylating 5‐azacytidine and pyrosequencing was performed. Combination miR‐100‐5p/miR‐125b‐5p mimic replenishment was used to treat malignant GCT cells. Global messenger RNA (mRNA) targets of the replenished miRNAs were identified and Metascape used to study pathway effects. We found that expression levels of miR‐99a‐5p/miR‐100‐5p and miR‐125b‐5p, their respective pri‐miRNAs, and associated genes from chromosomes 11 and 21 (chr11/chr21) were downregulated and highly correlated in malignant GCT cells. Treatment with 5‐azacytidine caused upregulation of these miRNAs, with pyrosequencing revealing hypermethylation of their chr11/chr21 loci, likely contributing to miR‐100‐5p/miR‐125b‐5p downregulation. Combination miR‐100‐5p/miR‐125b‐5p mimic replenishment resulted in growth inhibition in Sem/YST cells, with miR‐100‐5p/miR‐125b‐5p mRNA targets enriched in downregulated genes, which were involved in cell cycle (confirmed by flow cytometry) and signaling pathways. Knockdown of the miR‐100‐5p/miR‐125b‐5p target *tripartite motif containing 71* (*TRIM71*kd) recapitulated miR‐100‐5p/miR‐125b‐5p replenishment, with growth inhibition and cell cycle disruption of Sem/YST/EC cells. Further, replenishment led to reduced *lin‐28 homolog A* (*LIN28A*) levels and concomitant increases in *let‐7* (*MIRLET7B*) tumor suppressor miRNAs, creating a sustained reversion of cell phenotype. In summary, combination miR‐100‐5p/miR‐125b‐5p mimic replenishment or *TRIM71*kd caused growth inhibition in malignant GCT cells via cell cycle disruption. Further studies are now warranted, including mimic treatment alongside conventional platinum‐based chemotherapy.

## Graphical abstract

MiR‐99a‐5p/miR‐100‐5p (functionally identical) and miR‐125b‐5p microRNAs are downregulated in malignant germ cell tumors (GCTs). Combination replenishment of these microRNAs using mimics resulted in growth inhibition in representative cell lines, with consequent downregulation of target genes involved in cell cycle (confirmed by flow cytometry) and signaling pathways. Further studies are now warranted, including mimic treatment alongside conventional platinum‐based chemotherapy.

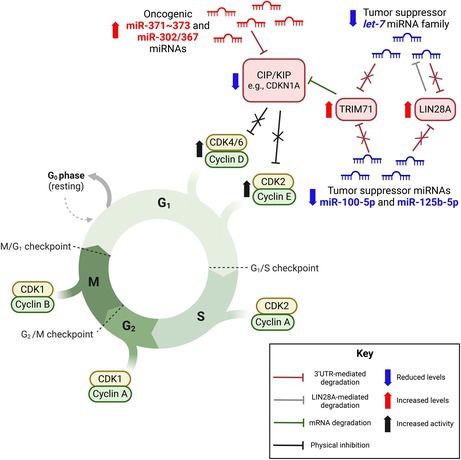

**Abbreviations**
3′UTR: 3′ untranslated region
5′aza: 5′azacytidine
ATCC: American Type Culture Collection
BSP: bisulphite‐converted DNA‐specific PCR
CCLG: Children's Cancer and Leukaemia Group
CHC: choriocarcinoma
chr: chromosome
CL: cell lines
CPDV: change‐point delta value
d: day
DNA: deoxyribonucleic acid
EC: embryonal carcinoma
EVs: extracellular vesicles
FDR: false discovery rate
GCNIS: germ cell neoplasia *in situ*
GCT: germ cell tumor
gDNA: genomic DNA
GTEx: Genotype‐Tissue Expression
kd: knockdown
kDa: kilodaltons
lincRNA: long intergenic noncoding RNA
M: median
miRNA: microRNA
MNC: mimic‐negative‐control‐#1
mRNA: messenger RNA
*n*
: number
NS: nonsignificant
NSGCT: nonseminomatous GCT
nt: nucleotide
NTC: nontargeting control
PCR: polymerase chain reaction
PGC: primordial germ cell
pri‐miRNA: primary miRNA
qRT‐PCR: quantitative reverse transcription polymerase chain reaction
RIN: RNA integrity value
RNA: ribonucleic acid
ROI: region of interest
RRID: Research Resource Identifier
RT: reverse transcription
SCR: seed complementary region
Sem: seminoma
SEM: standard error of the mean
siRNA: short interfering RNA
SSSS: single summed significance score
Ter: teratoma
YST: yolk sac tumor

## 1 Introduction

Germ cell tumors (GCTs) are a heterogeneous group of benign and malignant neoplasms that vary by clinical presentation, clinical course, and histology [1]. Despite their extensive clinical and pathological heterogeneity, all malignant GCTs are believed to originate from totipotent primordial germ cells (PGCs) [2]. Teratomas are typically benign tumors that display somatic differentiation [1]. Malignant subtypes are divided into germinomas [seminoma in the testis, dysgerminoma in the ovary, and germinoma at other extragonadal sites; subsequently referred to throughout as seminoma (Sem)] and nonseminomatous tumors [NSGCTs; embryonal carcinoma (EC), yolk sac tumor (YST), and choriocarcinoma (CHC)]. Differentiation status in malignant subtypes varies from Sem, which retain pluripotent, undifferentiated characteristics, through EC, which demonstrate early embryonal differentiation, to other NSGCTs such as YST and CHC, which show extraembryonic differentiation characteristic of the yolk sac and trophoblast, respectively [3].

Although most patients with malignant GCTs have a good prognosis since the introduction of platinum‐based chemotherapy, some still experience inferior outcomes, either in terms of survival or quality of life [3]. Late effects of treatment include ototoxicity [4] and nephrotoxicity [5], myelosuppression [6], pulmonary toxicity [7], cardiovascular disease [8], and second malignant neoplasms [9]. Furthermore, testicular germ cell malignancy remains a leading cause of death in young men [10]. Improved understanding of the molecular pathogenesis of malignant GCTs would therefore represent an important step towards developing novel therapeutic agents with favorable toxicity profiles, which may improve survival for patients with high‐risk disease and reduce toxicity for low‐risk patients [3]. Identifying such molecular abnormalities that are shared across the diverse malignant GCT spectrum is of particular importance, as these are likely to fundamentally contribute to disease pathogenesis [1]. Identification of novel targets and the development of more targeted and less toxic therapies for patients with malignant GCTs therefore remains an unmet clinical need. Such an approach will be key to improving outcomes for patients with malignant GCTs, particularly given the young median age when these tumors arise [11].

MicroRNAs (miRNAs) are short, nonprotein‐coding RNAs, typically 21–23 nucleotide (nt) in length, that post‐transcriptionally regulate protein‐coding gene expression via their 5′ ‘seed’ region, binding the corresponding seed complementary region (SCR) in the 3′ untranslated region (3′UTR) of their mRNA targets [12]. They are dysregulated in cancer, with most miRNAs being downregulated [13]. Consistent with these observations, we identified that most miRNAs were downregulated in malignant GCTs when compared with nonmalignant control tissues (comprising normal gonadal tissues and teratomas) [1]. The three top‐ranking downregulated putative tumor suppressor miRNAs in this study were miR‐99a‐5p, miR‐100‐5p, and miR‐125b‐5p [1]. These miRNAs are transcribed as polycistrons from two miRNA clusters, resulting in similar expression levels. The mature form of miR‐125b‐5p is the product of two genomic loci, each of which is physically clustered with either miR‐99a‐5p or miR‐100‐5p. The miR‐100~miR‐125b‐1 cluster is encoded on chromosome 11q24.1, while the miR‐99a~miR‐125b‐2 cluster is encoded on chromosome 21q21.1 [14]. Of note, both miR‐125b‐5p and miR‐100‐5p are dysregulated by epigenetic mechanisms related to promoter hypermethylation in several other cancer types, for example, [15, 16], with associated tumor suppressor function [17]. Furthermore, clustered miRNAs have been shown to exert combined effects, demonstrated, for example, by miR‐144/451 regulation of erythroid development [18]. We have previously highlighted the potential for miRNA replenishment strategies in malignant GCTs and their expected minimal off‐target effects [19], and an early‐phase clinical trial involving miRNA replenishment therapy for cancer in humans has reported [20]. However, to date, the upstream regulation, and downstream consequences, of miR‐99a‐5p, miR‐100‐5p, and miR‐125b‐5p downregulation have not been systematically explored in malignant GCTs. Here, we demonstrate that promoter hypermethylation is likely to contribute to the downregulation observed in malignant GCT tissues and that combined replenishment results in sustained phenotypic and genotypic changes *in vitro*. This work provides an essential platform for the *in vivo* studies that are now warranted to explore further the potential of miRNA replenishment therapy in malignant GCTs.

## 2 Materials and methods

### 2.1 Patient samples

The study was performed under multicenter generic Children's Cancer and Leukaemia Group (CCLG) Tissue Bank approval (East‐Midlands/Derby REC reference 08/h0405/22 + 5, covering Biological Studies CCLG‐2002‐BS03 and CCLG‐2020‐BS02; formerly Trent‐REC reference 02/4/071) and Cambridge Local Research Ethics Committee (reference 01/128) approval. Written informed consent was obtained from all subjects. The study methodology conformed to the standards set by the Declaration of Helsinki. Samples for this study from the CCLG Tissue Bank were collected between January 1987 and October 2005, via 21 Principal Treatment Centre institutions (https://www.cclg.org.uk/In‐hospital/Specialist‐hospitals). Reanalysis of published microarray miRNA expression profiling data [1, 21] was undertaken on 42 clinical GCT samples, comprising 32 pediatric GCTs from 22 female and 10 male patients (12 YSTs, 11 Sem, three ECs and six teratomas), two testicular Sem from young adults and eight control samples, consisting of two prepubertal normal gonadal specimens (one from a female and one from a male patient), two postpubertal normal gonadal specimens (one from a female and one from a male patient), and four developmental samples (two fetal yolk sacs and two fetal female gonads), as described [22]. Due to their extreme rarity and diagnosis often based on elevated serum human chorionic gonadotropin levels rather than biopsy, no CHC samples were available for study. A partially independent set of 24 clinical GCT samples (nine YSTs, eight Sem, two EC, and five teratomas) and two gonadal control samples using total RNA from human ovaries and testes (AM6974/AM7972, respectively; Ambion, now Thermo Fisher Scientific, Bishop's Stortford, UK) was used for qRT‐PCR validation of microarray miRNA expression findings [21]. As the majority of GCTs are gonadal, these Ambion samples were used as a pooled gonadal control for gene expression studies to allow comparison with GCT tissues and cell lines, as described [21]. Available clinicopathological information for these samples, obtained from the biobanks from which they were received, is listed in Table S1. Additionally, GCT cell lines were used for microarray (*n* = 6) and qRT‐PCR (*n* = 7) analyses (Section 2.2). Finally, messenger RNA (mRNA) array data for 45 clinical samples, comprising 37 malignant GCTs (17 pediatric, 20 adult) and eight nonmalignant controls (three pediatric, five adult) [1, 21] was used for clinical correlation of functional investigations in cell lines, as described [22]. This array data is publicly available at Gene Expression Omnibus, accession no. GSE18155.

### 2.2 GCT cell lines

For *in vitro* studies, we used human malignant GCT cell lines representing the range of common histological subtypes encountered in clinical practice, namely Sem, YST, and EC, as described [21, 22, 23]. These were 2102Ep (EC) [ExPASy Cellosaurus online cell line knowledge resource (https://web.expasy.org/cellosaurus/) Research Resource Identifier (RRID): CVCL_C522] [24] and 1411H (RRID: CVCL_2268) [25] from P. Andrews, University of Sheffield, UK; and GCT44 (RRID: CVCL_A346) [26] (both YST) and TCam2 (Sem) (RRID: CVCL_T012) [27] from J. Shipley, Institute of Cancer Research, Marsden, Surrey, UK. It should be noted that no genuine GCT‐derived CHC cell lines are available for study [22]. Further analysis of published microarray miRNA expression profiling data was undertaken on six GCT cell lines [namely TCam2 (Sem), 1411H (YST), GCT44 (YST), 2102Ep (EC), Tera‐2 (EC/teratoma; RRID: CVCL_2777), and PA‐1 (immature teratoma; RRID: CVCL_0479)], as described [1, 21, 22]. In addition, NCCIT (EC) cells (RRID: CVCL_1451) were also used for qRT‐PCR validation. Tera‐2, PA‐1, and NCCIT cell lines were all obtained from the American Type Culture Collection (ATCC, Manassas, VA, USA). All cells were cultured at 37 °C in 5% CO_2_ in appropriate medium containing 10% fetal bovine serum (FBS) and 1% penicillin/streptomycin, as described [21, 22, 23], unless otherwise stated. All cell lines were authenticated by short tandem repeat (STR) profiling [28] within the last 3 years, and all experiments performed with mycoplasma‐free cells.

### 2.3 Quantitative reverse transcription PCR (qRT‐PCR) for miRNAs and primary miRNAs (pri‐miRNAs)

MiRNA and pri‐miRNA species were isolated from GCT cell lines using Tri Reagent (Sigma‐Aldrich, now Merck Life Science UK Ltd, Watford, UK), following the TRIzol RNA extraction protocol, as described [21]. Levels of miRNAs were quantified in triplicate using TaqMan qRT‐PCR assays (Applied Biosystems, Warrington, UK) in two separate steps, namely reverse transcription (RT) followed by PCR quantification, as per the manufacturer's instructions, and normalized to *RNU24* (*small nucleolar RNA*, *C/D box 24* or *SNORD24*), as described [1, 21, 22]. As the sequences of miR‐99a‐5p (AACCCGUAGAUCCGAUCUUGUG) and miR‐100‐5p (AACCCGUAGAUCCGAACUUGUG) differed by only a single (underlined) nt towards the 3′ end, and thus represent a functional ‘unit’ with regards their seed region and mRNA targets, we used miRNA expression levels using the miR‐100‐5p primer/probe set as a surrogate for overall miR‐99a‐5p/miR‐100‐5p levels. Levels of pri‐miRNAs were quantified in quadruplicate using TURBO DNA‐free kit, TaqMan High‐Capacity cDNA Reverse Transcription kit, and TaqMan qRT‐PCR assays (all Applied Biosystems), according to the manufacturer's instructions (apart from using higher total RNA input of 100 ng due to low pri‐miRNA abundance) and normalized to *50S ribosomal protein L15* (*RPLO*), *glucuronidase beta* (*GUSB*), and the ribosomal RNA *18S* [21]. Expression levels were quantified using the delta Ct method and referenced to a pooled gonadal control sample composed of equal amounts of total RNA from human ovaries and testes (AM6974/AM7972, respectively; Ambion, now Thermo Fisher Scientific), as described [21].

### 2.4 Genomic copy number determination across the miR‐99a‐5p/miR‐100‐5p and miR‐125b‐5p loci on chromosomes 11 and 21

Primers to assess genomic copy number were designed using the website ‘Primer3’ (https://bioinfo.ut.ee/primer3/) [29], as described [22] (Table S2). Quantitative PCR was performed on genomic DNA (gDNA) extracted from the four cell lines TCam2, 1411H, GCT44, and 2102Ep using standard phenol:chloroform extraction, with levels normalized to four gDNA housekeeping regions [*beta‐2‐microglobulin* (*B2M*), *glyceraldehyde‐3‐phosphate dehydrogenase* (*GAPDH*), and two genomic regions on chromosome 18 (termed ‘18A’ and *‘*18B’)], all regions not genomically altered in malignant GCTs, and compared with pooled gonadal control using human ovarian and testicular gDNA (Thermo Fisher Scientific) levels.

### 2.5 Quantification of methylation levels upstream of the miR‐99a‐5p/miR‐100‐5p and miR‐125b‐5p loci on chromosomes 11 and 21

In initial proof‐of‐principle screening, malignant GCT cells were treated with 5 μm of the demethylating agent 5‐azacytidine (5′aza; Sigma‐Aldrich), and relevant miRNA, long intergenic noncoding RNA (lincRNA) and protein‐coding gene expression quantified on day (d) 3 and d4. Next, gDNA was isolated using phenol:chloroform from malignant GCT cells, control gDNA from normal adult testis tissue (Catalogue: R1234260‐50; BioChain Institute, Newark, CA, USA), control samples from three primary cultures of normal cervical squamous epithelium (95:14, 95:15, and NCX6) [30] available in our laboratory, the normal fibroblast cell line HFFF2 (RRID: CVCL_2489), and the retinal pigment epithelium cell line 340‐RPE‐11tv (CVCL_W078) (both gifts from within the Department of Pathology, Cambridge, UK).

The accurate identification of promoter regions for miRNAs, where methylation typically occurs, is difficult and remains challenging [31]. Accordingly, we screened for potential CpG islands in the region up to 10 kb upstream of the transcriptional start of the lincRNA transcripts on both chromosomes 11 (chr11) [*MIR100HG*, including the protein‐coding gene *BH3‐like motif containing, cell death inducer* (*BLID*); 11q24.1] and 21 (chr21) (*MIR99AHG*, also known as *LINC00478*; 21q21.1) from where miR‐99a/miR‐100 and miR‐125b miRNAs are transcribed, were identified using methprimer (https://www.urogene.org/methprimer/) [32]. For this work, a minimum GC content cutoff of 50% was used to define a potential CpG island in the regions of interest (ROIs). In addition to predicting potential CpG islands in DNA sequences, methprimer designs bisulphite‐converted DNA‐specific PCR (BSP) primers within, or flanking, the CpG islands of interest. Initial PCR was thus performed to screen these ROIs (Table S3). PCR amplification of the resultant selected ROIs on chr11 and chr21 was performed in triplicate using SYBR Green JumpStart Taq ReadyMix (Sigma‐Aldrich), using methprimer designed BSP primers. Melting curve analysis was used to determine areas of potential differential methylation, as described [33], comparing results for malignant GCT cell lines with those obtained for normal gonadal tissue, and these areas were then further amplified using biotinylated primers to generate template for pyrosequencing. Sequencing was performed on a PyroMark‐Q24 pyrosequencer, using primers designed using pyroq software (Qiagen, Manchester, UK) (Table S4), and standard protocols, as per the manufacturer's instructions. For each cell line or tissue sample used, assays were performed in duplicate on a minimum of three independently prepared bisulphite‐converted gDNA samples. Cumulative abundance of CpG methylation was calculated by adding the percentage of methylation at each of the analyzed sites, assigning a value of 0 to unmethylated sites and 100 to fully methylated sites. In addition to this screening approach, a published global methylation study in malignant GCT cell lines and tissues [34] using the Illumina HumanMethylation450 BeadChip was also interrogated for further supportive evidence of hypermethylation. For this work, the 100 kb regions upstream of the ROIs on chr11 and chr21 were studied, with a minimal requirement for five probes within each of these regions. The ROI on chr21 only contained four probes and was not considered further, whereas chr11 contained seven probes, for which beta values and median (*M*) values were derived and compared with values in teratomas as a control. *M* values per probe were calculated using the formula: *M* value = log_2_(beta/1 − beta).

### 2.6 Cell transfection and proliferation assays

Following optimization of transfection conditions, cells were seeded into 6‐well plates with antibiotic‐free media and incubated for 24 h to ~ 50% confluency, at which point transfection was undertaken. 2102Ep cells were seeded at 7.5 × 10^5^ per plate, while TCam2, 1411H, and GCT44 cells were seeded at 1 × 10^5^ per plate. Cells were transfected with synthetic miR‐100‐5p or miR‐125b‐5p mirVana mimics (assay number: MC10188/MC10148, respectively), either alone or in combination, or with miRNA mimic‐negative‐control‐#1 (MNC; catalogue 4464059) (all Thermo Fisher Scientific). Given the virtually identical sequences of miR‐99a‐5p and miR‐100‐5p, and their identical 2–7 nt seed region, miR‐100‐5p mimic treatment served to functionally replenish both miR‐99a‐5p and miR‐100‐5p. Transfection was performed using Gibco Opti‐MEM media (Thermo Fisher Scientific) and the transfection reagent Lipofectamine‐RNAiMAX (Thermo Fisher Scientific), as described [21]. For ‘combination’ cotransfection of miR‐100‐5p and miR‐125b‐5p, each mimic was added at a concentration of 8.33 nm (combined concentration 16.7 nm) [18] and compared with cells transfected with an equimolar (16.7 nm) concentration of MNC. The optimal length of mimic transfection, which minimized toxicity and maximized transfection efficiency [21], was 8 h for 2102Ep, 6 h for 1411H and GCT44, and 4 h for TCam2. For *tripartite motif containing 71* (*TRIM71*) knockdown (*TRIM71*kd), cells were seeded as described above, except 2102Ep at 2 × 10^5^ per plate, and treated with *TRIM71* ON‐TARGETplus pool short interfering RNA (siRNA) (catalogue L‐023459‐01‐0005; Dharmacon, Lafayette, CO, USA) or nontargeting control (NTC) ON‐TARGETplus pool siRNA (catalogue D‐001810‐10‐05; Dharmacon) at 40 nm concentration for 6 h, using the transfection media and reagents described above. Experiments were performed in biological triplicate for individual treatment conditions, each with technical replicates. Cells were harvested for further study at set timepoints until d7. Cell numbers (live cell counts) were quantified using Trypan blue on a Countess automated‐cell‐counter (Invitrogen, Paisley, UK), determining the mean of two values for each of three replicates and then taking the mean of the resulting three values, as described [21, 22, 23].

### 2.7 Messenger RNA (mRNA) microarray analysis of combination miR‐100‐5p and miR‐125b‐5p‐treated cells on d2 and d7

We undertook global mRNA microarray profiling of TCam2, 1411H, and 2102Ep cells, comparing cells treated with combination miR‐100‐5p and miR‐125b‐5p mimics (total concentration 16.7 nm) with controls. At d7 after treatment, 24 triplicate samples were profiled, comprising TCam2, 1411H, and 2102Ep cell lines receiving combination treatment (*n* = 9 samples) and MNC treatment (*n* = 9), plus untreated cells for TCam2 and 1411H (*n* = 6). Importantly, no substantial differences in mRNA gene expression were observed when comparing the d7 MNC and respective untreated cells, highlighting the suitability of MNC‐treated cells to act as the control comparison for mimic‐treated cell conditions (data available at EBI Array Express, see below). At d2 after treatment, 12 triplicate samples were profiled, comprising TCam2 and 1411H cells receiving combination treatment (*n* = 6) and MNC treatment (*n* = 6). 2102Ep cells were excluded from d2 global mRNA profiling due to the absence of phenotypic effects at d7 post‐replenishment. Microarray experiments were performed at Cambridge Genomic Services, University of Cambridge. In brief, 200 ng of RNA underwent linear amplification using the Illumina TotalPrep RNA Amplification Kit (Life Technologies, Paisley, UK), as per the manufacturer's instructions. All samples had RNA integrity numbers (RIN) > 9.0. Copy RNA was hybridized overnight to HumanHT‐12‐v4 BeadChips (Illumina, Cambridge, UK), followed by washing, staining, and scanning using the BeadArray Reader (Illumina). Data were processed using the bioconductor packages lumi [35] and limma [36] in r. Across all samples, array probes for which the intensity values were not significantly different (*P* > 0.01) from the negative controls were removed (number of remaining probes: range 18 930–19 749 for different experimental conditions). For these probes, detailed full‐length 3′UTR information was available from the APPRIS database (http://appris.bioinfo.cnio.es) for 8702–9004 genes (Table S5). Following these steps, comparisons were performed using the limma package, with a false discovery rate (FDR) correction applied to account for multiple testing [37], using a significance cutoff of 0.1. Raw microarray data from these analyses is deposited at the EBI Array Express (https://www.ebi.ac.uk/biostudies/arrayexpress), accession no. E‐MTAB‐13464.

### 2.8 *Sylamer* analysis

As each miRNA can target hundreds of mRNAs, changes in individual mRNA expression levels following miRNA perturbation experiments are very subtle [38] and thus best assessed by global and pathway analyses, as described [22]. The *Sylamer* algorithm was therefore used to assess for global enrichment and/or depletion of nucleotide ‘words’ of specific length (SCRs), complementary to elements of miRNA seed regions (nucleotide positions 1–8) of interest within the 3′UTRs of genes within ranked lists [39], derived from miRNA replenishment and MNC‐treated cells. The output was visualized as a landscape plot of *P*‐values for each SCR (*y*‐axis), plotted against the ranked gene list (*x*‐axis), and segregated into ‘bins’ containing 200 genes in each [1, 39]. The derived single summed significance score (SSSS) was an integration of *Sylamer* significance scores for different elements that comprised the SCR and served as an overall evaluation of the enrichment or depletion of nucleotide sequences [21]. For this work, the scores were calculated by combining the *Sylamer* results for six SCR elements, all complementary to the 1–8 nt seed region of miR‐100‐5p or miR‐125b‐5p (Table S6). Following generation of SSSS landscape plots, a change‐point detection algorithm was employed to identify the most appropriate enrichment peak for selecting gene lists for further analyses, as described [22]. In brief, this algorithm computed a change‐point delta value (CPDV) for each bin of 200 genes, based on the difference between its −log_10_(*P*‐value) and the minimum −log_10_(*P*‐value) across the next five bins (progressing from left to right). CPDV curves were plotted for the SCR of interest (corresponding to the 1–8 nt miRNA seed of interest) and the bin with the maximum CPDV selected [22].

### 2.9 Metascape pathway analysis

Global mRNA data were used to study effects on protein‐coding gene expression and cellular pathways. Gene and 3′UTR information was downloaded from Ensembl, using the bioconductor r package biomart, as described [23]. In brief, the SCR‐containing downregulated gene lists identified using the CPDV algorithm and derived from combination miRNA replenishment treated cell lines, underwent pathway analysis using metascape software (https://metascape.org/) [23], using version v3.5.20230101, as described [23]. Pathway terms were ranked by *P*‐value and used to select pathways for further analyses, based on known cellular and biological functions. In addition, for global mRNA data from clinical samples, we conversely selected upregulated (de‐repressed) targets in malignant GCT cases (log_2_ fold change > 0.5), from which 114 miR‐100‐5p targets and 683 miR‐125b‐5p targets were identified (total 797 targets), with an overlap of 63 genes. In total therefore, 734 unique miR‐100‐5p or miR‐125b‐5p SCR‐containing upregulated gene targets were identified, which were analyzed using metascape.

### 2.10 Western blotting

Western blots were conducted for TRIM71 and LIN28A [21] proteins using antibodies sc‐393352 (1 : 300; Santa Cruz Biotechnology, Wembley, UK) and ab46020 (1 : 5000; Abcam, Cambridge, UK), respectively. Results were normalized to TUBB (‘tubulin’; ab6046; 1 : 10 000; Abcam). Images were scanned and densitometry analyses were performed using fluorchem‐9900 imaging system software (Alpha Innotech, San Leandro, CA, USA), with imagej software (https://imagej.net) used to quantify protein expression, as described [21].

### 2.11 Flow cytometry assay

Cell cycle analysis was performed at d2 and d3 following transfection using Click‐iT‐EdU‐Alexa‐Fluor‐647 Flow Cytometry Assay Kit (Thermo Fisher Scientific), as per the manufacturer's instructions, and as described [22]. For this work, TCam2 (Sem) and 2102Ep (EC) cells were used where indicated, as 1411H (YST) cells did not adequately incorporate EdU dye [22]. In brief, 1 μL of FxCycle Violet (Thermo Fisher Scientific) was then added to the final mixture to stain cellular DNA and samples analyzed using a flow cytometer BD‐LSR‐Fortessa machine [Beckton Dickinson (BD) Biosciences, Wokingham, UK] at the Wellcome‐MRC Cambridge Stem Cell Institute. For the detection of EdU dye with Alexa‐Fluor‐647, azide 633/635 nm excitation with a red emission filter (660/20 nm) was used. Flow cytometer data were analyzed in biological triplicate using flowjo (FlowJo LLC, BD Biosciences) (version 10.5.0).

### 2.12 Statistics

Statistical analyses were performed using graphpad prism 6 software (GraphPad Software, La Jolla, CA, USA). For comparisons between groups, unpaired two‐tailed Student's *t*‐test were used unless otherwise stated; *P*‐values ≤ 0.05 were considered statistically significant. Data presented represent mean values ± standard error of the mean (SEM) unless otherwise stated.

## 3 Results

### 3.1 Downregulation of miR‐99a‐5p, miR‐100‐5p, and miR‐125b‐5p (and overall 2–7 nt miRNA seed abundance) is universally observed in malignant germ cell tumor (GCT) clinical samples and cell lines

**Fig. 1:**
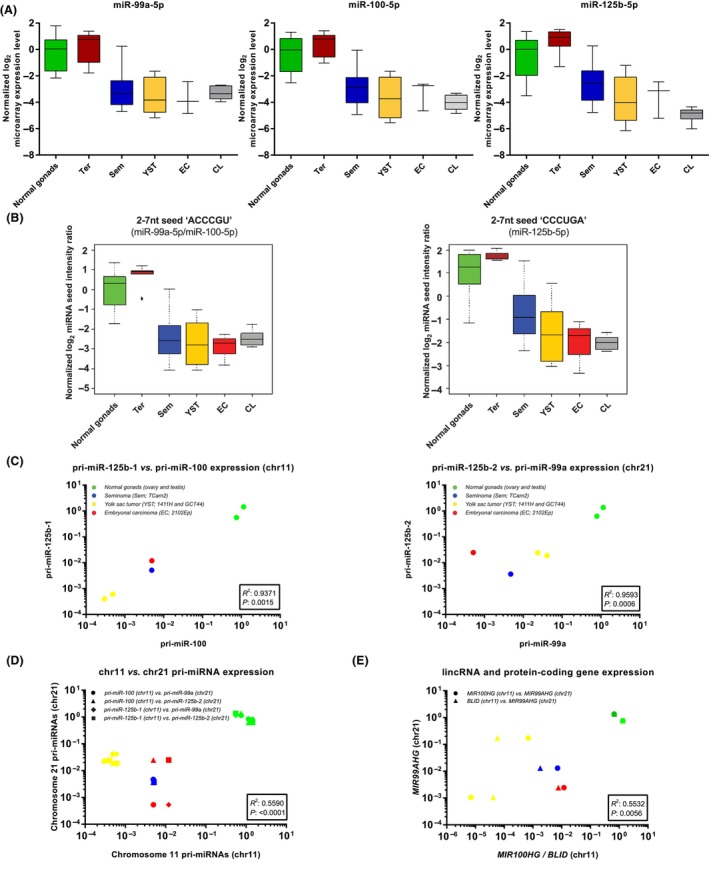
MiR‐99a‐5p, miR‐100‐5p, and miR‐125b‐5p expression and correlations of primary microRNA (pri‐miRNA), long interspersed noncoding RNA (lincRNA), and protein‐coding gene expression from chromosomes 11 and 21 in malignant germ cell tumor (GCT) clinical samples and cell lines relative to nonmalignant (gonadal and teratoma) control samples. (A) Normalized log_2_ microarray expression levels for miR‐99a‐5p, miR‐100‐5p, and miR‐125b‐5p. (B) Summated normalized log_2_ microarray intensity ratio boxplots showing seed intensity for the nucleotide (nt) sequences ‘ACCCGU’ (left; 2‐7nt seed of miR‐99a‐5p/miR‐100‐5p) and ‘CCCUGA’ (right; 2–7 nt seed of miR‐125b‐5p). The clinical samples in (A) and (B) represent normal gonads (*n* = 8, green), teratomas (Ter; *n* = 5, brown), seminoma (Sem; *n* = 13, blue), yolk sac tumor (YST; *n* = 12, yellow), embryonal carcinoma (EC; *n* = 3, red), and malignant GCT cell lines (CL; *n* = 6, gray). In all panels in (A) and (B), the horizontal bar = median expression and the box = minimum to maximum values with whiskers = standard deviation. It should be noted that it is not possible to reliably apply statistical testing to these normalized log_2_ microarray expression levels in (A) nor the derived intensity ratios in (B). (C) Correlations between expression of pri‐miRNAs from chromosome 11 (chr11; left panel; pri‐miR‐125b‐1 *vs*. pri‐miR‐100) and chromosome 21 (chr21; right panel; pri‐miR‐125b‐2 *vs*. pri‐miR‐99a). (D) Correlation between pri‐miRNA expression from chr11 and chr21. (E) Correlation between expression of associated lincRNA/protein‐coding genes on chr11 (*MIR100HG/BLID*) and chr21 (*MIR99AHG*), which include the regions encoding the miRNAs of interest. In (D, E), the comparison for each gene type is indicated by a symbol (circle, triangle, square, diamond), as described in the Figure. All expression values are referenced to the mean of pooled normal gonadal (ovary/testis) control samples. Color‐coding for (D) and (E) is as described in the color key (C). For experiments (C–E), statistical significance is determined using linear regression analysis.

Microarray data analysis demonstrated substantial and significant downregulation of miR‐99a‐5p, miR‐100‐5p, and miR‐125b‐5p across all malignant GCT tissue samples and cell lines when compared with nonmalignant (gonadal and teratoma) control samples (Fig. 1A, Table S7). Further analyses confirmed downregulation of miR‐99a‐5p, miR‐100‐5p, and miR‐125b‐5p in each of the malignant GCT subtypes, namely germinoma/seminoma (Sem), yolk sac tumor (YST), and embryonal carcinoma (EC), and in the panel of representative GCT cell lines proposed for *in vitro* studies (Table S7).

Microarray data analysis demonstrated that the normalized intensity ratios for miRNAs containing the 2–7 nt seed ‘ACCCGU’ of miR‐99a‐5p/miR‐100‐5p (and also miR‐99b‐5p, which is transcribed separately from chromosome 19 at 19q13.41), and 2–7 nt seed ‘CCCUGA’ of miR‐125b‐5p (and also miR‐125a‐5p, again transcribed from 19q13.41), were substantially lower in malignant GCT samples and cell lines when compared with nonmalignant control tissues, comprising gonadal and teratoma tissues (Fig. 1B). Of note, miR‐99b‐5p and miR‐125a‐5p were also generally downregulated in malignant GCTs, but not so consistently or substantially as miR‐99a‐5p/miR‐100‐5p and miR‐125b‐5p (Table S7), providing further evidence for pursuing miR‐99a‐5p/miR‐100‐5p and miR‐125b‐5p experimentally. Accordingly, using qRT‐PCR validation, expression levels of miR‐99a‐5p/miR‐100‐5p and miR‐125b‐5p were confirmed as downregulated in malignant GCT clinical samples and cell lines, compared with nonmalignant (gonadal and teratoma) controls, and showed a very strong correlation with each other by linear regression analysis (*R* ^2^ = 0.965; *P* < 0.0001) (Fig. S1), confirming likely coordinated downregulation of both miRNA clusters in malignant GCTs. In terms of basal expression levels of these miRNAs, qRT‐PCR levels seen in malignant GCT tissues were typically ~ 1–20% of those observed in the nonmalignant control group and for malignant GCT cell lines, ~ 0.01–1% (Fig. S1), demonstrating their suitability for subsequent replenishment studies.

### 3.2 The miR‐99a‐5p/miR‐100‐5p and miR‐125b‐5p loci on chromosomes 11 and 21 are transcriptionally downregulated in malignant GCT cells

To address the mechanism of miR‐99a‐5p/miR‐100‐5p and miR‐125b‐5p downregulation, we first quantified levels of the respective primary miRNA transcripts (pri‐miRNAs) in four representative malignant GCT cell lines. In all cases, levels of transcription were reduced compared with pooled gonadal control (*P* < 0.0001) (Fig. S2A,B). There was a very strong correlation between levels of the primary transcripts derived from the chr11 locus (11q24.1; pri‐miR‐100 and pri‐miR‐125b‐1) (*R* ^2^ = 0.94 and *P* = 0.0015) (Fig. 1C) and also the chr21 locus (21q21.1; pri‐miR‐99a and miR‐125b‐2) (*R* ^2^ = 0.96 and *P* = 0.0006) (Fig. 1C). When levels of each chr11 pri‐miRNA were compared with each chr21 pri‐mRNA (i.e., four comparisons in total), the combined data showed a significant correlation in expression from the two loci (*R* ^2^ = 0.559; *P* < 0.0001) (Fig. 1D). Moreover, other genes which co‐localized with the miRNA clusters (namely *MIR100HG* and *BLID* on chr11; *MIR99AHG* on chr 21) also showed reduced expression levels in all four cell lines compared with pooled gonadal control (*P* < 0.0001) (Fig. S2C). Finally, when levels of each chr11 gene were compared with levels of *MIR99AHG* (i.e., two comparisons), the combined data showed a significant correlation in the expression of the genes from the two chromosomal loci (*R* ^2^ = 0.553; *P* = 0.0056) (Fig. 1E). Together, these findings very strongly suggested that these miRNAs are coregulated and transcriptionally downregulated at the chr11 and chr21 clusters, together with neighboring lincRNA and protein‐coding genes.

### 3.3 No evidence of overall copy number loss to account for downregulation of miR‐99a‐5p/miR‐100‐5p and miR‐125b‐5p loci on chromosomes 11 and 21

Genomic copy number assessment showed no consistent evidence of loss across the relevant chr11 and chr21 loci in the malignant GCT cell lines interrogated relative to pooled gonadal control (Fig. S3) to account for the miRNA downregulation observed.

### 3.4 The chromosome 11 and 21 miRNA clusters show evidence of upstream hypermethylation in malignant GCT cells

**Fig. 2:**
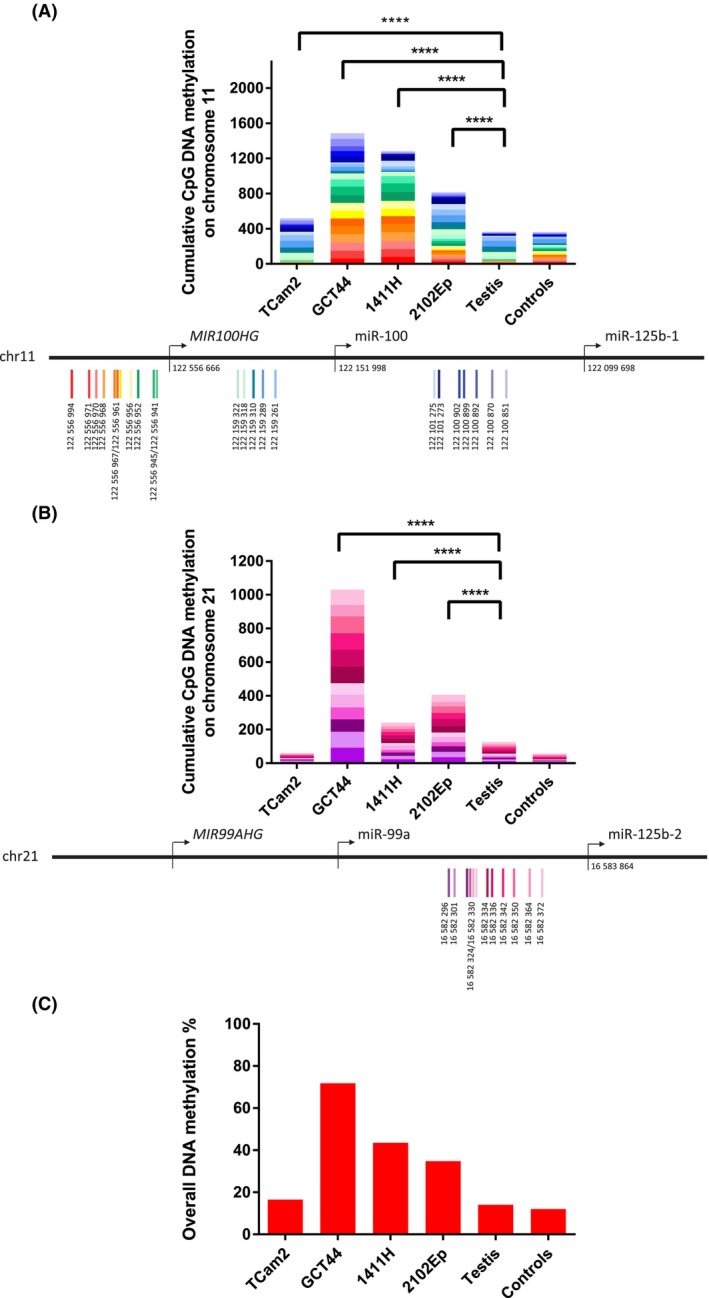
DNA methylation at chromosome 11 and 21 loci in malignant germ cell tumor (GCT) cells. In each panel in (A, B), the upper figure shows the cumulative levels of endogenous CpG DNA methylation across the chromosome 11 (chr11; A) or chromosome 21 (chr21; B) locus, while the lower figure represents the genomic location map of all the CpG sites examined. The colors in each bar (from the base to top) correspond to the individual CpG coordinates (from left to right) in the respective genomic map. The size of each bar represents the average percentage of methylation per CpG site, ranging from 0 (no methylation) to 100% (fully methylated). Accordingly, given 23 and 12 CpG sites assessed for chr11 and chr21, respectively, the maximum cumulative methylation score for each is 2300 and 1200. *P*‐values (Student's *t*‐test): ****P < 0.0001. The controls comprised five tissues/cell lines, namely three normal cervical squamous epithelium samples and the normal cell lines HFFF2 (fibroblasts) and 340‐RPE‐11tv (retina). (C) Overall DNA methylation percentage (%) for the malignant GCT cell lines, testis, and controls. The percentage given represents methylation across 35 CpG sites on both chr11 and chr21 and thus is out of a total cumulative methylation score of 3500.

We sought to establish whether hypermethylation contributed to reduced transcription from chr11 and chr21 loci in malignant GCT cells. Indeed, malignant GCT cells treated with 5‐azacytidine showed some upregulation at d3 and d4 for miR‐100‐5p and miR‐125b‐5p (Fig. S4A), as well as for lincRNAs *MIR100HG* (chr11) and *MIR99AHG* (chr21) and the protein‐coding gene *BLID* (chr11) (Fig. S4B), consistent with contributions from hypermethylation at these sites. Next, following these proof‐of‐principle 5‐azacytidine studies, we used pyrosequencing to assess methylation level within CpG islands at chr11 (four regions of interest; ROIs) and chr21 (one ROI) (Fig. S4C). There was significantly greater cumulative abundance of CpG methylation in the malignant GCT cell line group compared with the nonmalignant control group (comprising normal testis tissue and control cells) at both chr11 and chr21 loci (*P* < 1 × 10^−15^) (Fig. 2). Together, these data suggested that downregulation of miR‐99a‐5p/miR‐100‐5p and miR‐125b‐5p in malignant GCTs is associated with hypermethylation at both chr11 and chr21 from where the miRNAs are transcribed. Further, analysis of published global methylation data showed hypermethylation across both malignant GCT cell lines and tissue samples (Fig. 4D), supporting the pyrosequencing data.

### 3.5 Combination replenishment of miR‐100‐5p and miR‐125b‐5p in malignant GCT cells causes growth inhibition

**Fig. 3:**
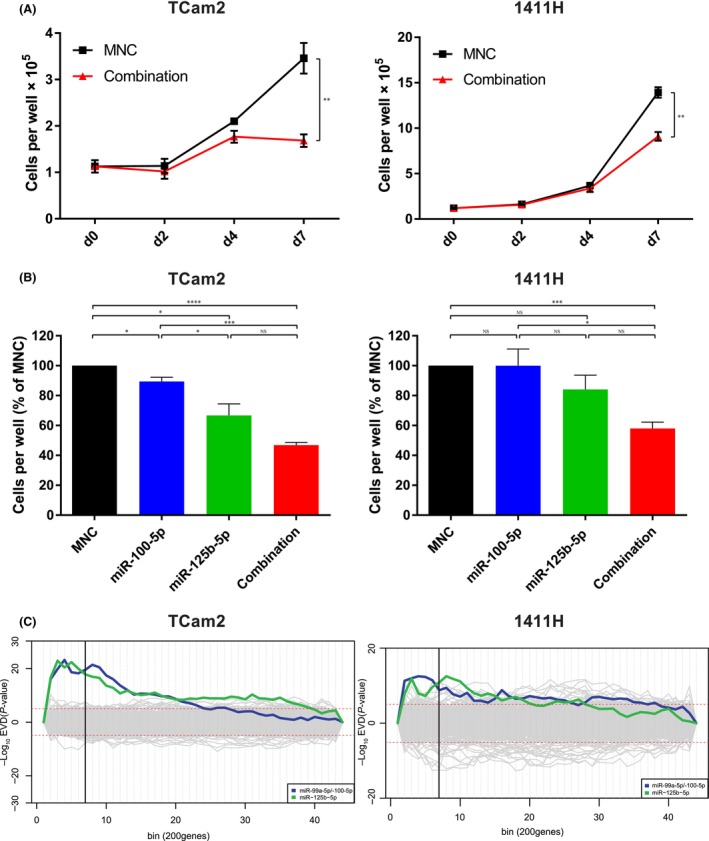
Phenotypic and genotypic effects of combination miR‐100‐5p and miR‐125b‐5p replenishment in malignant germ cell tumor (GCT) cells. (A) Growth curves for TCam2 (left) and 1411H (right) cells after transfection with total 16.7 nm concentration of combination miR‐100‐5p (8.33 nm) and miR‐125b‐5p (8.33 nm) mimics, or 16.7 nm mimic‐negative‐control (MNC). (B) TCam2 (left) and 1411H (right) cell number at day 7 (d7) after transfection with miR‐100‐5p and/or miR‐125b‐5p mimics, either alone or in combination, relative to MNC, which was assigned to 100%. All total concentrations used were 16.7 nm; where combination microRNA (miRNA) mimics were used, this was an 8.33 nm equimolar concentration of each. (C) *Sylamer* plots showing the single summed significance score (SSSS) of the seed complementary regions (SCRs) corresponding to miR‐99a‐5p/miR‐100‐5p (blue line) and miR‐125b‐5p (green line) in the ranked gene lists from TCam2 (left) and 1411H (right) cells at d2 following treatment with 16.7 nm combination miR‐100‐5p and miR‐125b‐5p mimic replenishment, compared with 16.7 nm MNC‐treated cells. Log_10_‐transformed *P*‐values for each SCR word are on the *y*‐axis, against the ranked gene list (from downregulated on the left to upregulated on the right) on the *x*‐axis. A positive *y*‐axis deflection on the left‐hand side of the plot signifies SCR enrichment in downregulated genes. Error bars = standard error of the mean (SEM). Statistical significance (Student's *t*‐test): *P ≤ 0.05, **P < 0.01, ***P < 0.005, ****P < 0.001; NS, nonsignificant. Note that all experiments were performed in biological triplicate (i.e., *n* = 3) for individual treatment conditions, each with technical replicates.

Next, to investigate the phenotypic and genotypic effects of miR‐99a‐5p/miR‐100‐5p and miR‐125b‐5p downregulation, levels were replenished in malignant GCT cell lines. In growth curves for TCam2 (Sem) and 1411H (YST) cells transfected with each mimic at 8.33 nm (16.7 nm total concentration), there was significant growth inhibition at d7 compared with cells transfected with mimic‐negative‐control‐#1 (MNC); *P* ≤ 0.01 for each cell line (Fig. 3A). In contrast, 2102Ep (EC) cells did not show growth inhibition using combination mimic replenishment at 16.7 nm, nor at an increased concentration of 33.3 nm (Fig. S5A). To interrogate this observation further, we next quantified the replenished miRNAs in recipient cells by qRT‐PCR, showing that levels of miR‐100‐5p and miR‐125b‐5p were substantially greater in both TCam2 and 1411H cells than in their 2102Ep counterparts at 16.7 nm (Fig. S5B). Of note, by d7, levels of both these miRNAs in TCam2 and 1411H were still ~ 1000–10 000‐fold higher than those in relevant MNC‐treated cells (Fig. 5B). Importantly, given these cell lines had basal levels as low as ~ 0.01% of normal controls (Fig. S1), observed levels represented likely physiological replenishment of these miRNAs. Next, we studied miRNA levels in extracellular vesicles (EVs), known to be released from malignant GCT cells and to be enriched in miRNA cargo [23]. We isolated EVs from 2102Ep and 1411H cells on d1 and d2 after treatment with 16.7 nm combination miRNA replenishment using ultracentrifugation, as described [23] and compared intracellular and EV miRNA levels. Both cell lines rapidly excreted the replenished miRNAs within EVs, predominantly within the first 24 h after transfection (Fig. S5C). However, whereas the EV miR‐100‐5p and miR‐125b‐5p levels relative to the intracellular levels for 1411H cells were 6.5% and 66.7% on d1, respectively, this figure was 1475% and 14 049%, respectively, for 2102Ep cells (Fig. S5C), providing a potential mechanistic explanation for the lack of phenotype observed in EC cells.

Next, we sought to confirm that the use of combination mimic replenishment was more significant than for either miRNA replenished individually, at the timepoint of maximal phenotypic difference (d7) in TCam2 and 1411H cells. As expected, growth inhibition in these cells was greater using the 16.7 nm combination (8.3 nm of each miRNA mimic) than with either mimic used alone at an equimolar concentration of 16.7 nm (Fig. 3B).

### 3.6 *Sylamer* analysis of global mRNA profiles in malignant GCT cell lines at d2 and d7 following combination replenishment with miR‐100‐5p and miR‐125b‐5p

Using the change‐point delta value (CPDV) method, bin 7 (Fig. S6), corresponding to the 1400 most downregulated genes, was identified for both TCam2 and 1411H as the optimal d2 cut point. To facilitate direct comparison between d2 and d7 data, the 1400 most downregulated genes were also selected for both TCam2 and 1411H in d7 analyses. Using *Sylamer*, there was highly significant enrichment for the seed complementary regions (SCRs) corresponding to the 1–8 nt seed region of both miR‐99a‐5p/miR‐100‐5p and miR‐125b‐5p in the most downregulated genes at d2 following combination mimic treatment, a timepoint at which the direct effects of miRNAs on mRNA transcripts are greatest [40], in both TCam2 and 1411H cells, shown in landscape plots (Fig. 3C). Enrichment for miR‐99a‐5p/miR‐100‐5p and miR‐125b‐5p persisted to d7 in both TCam2 and 1411H cell lines, the timepoint when significant growth inhibition was seen, again shown in landscape plots (Fig. S7). However, as expected, such genotypic changes were not present in 2102Ep EC cells at d7 (Fig. S7), consistent with the lack of growth phenotype observed.

At d2, the SCRs for miR‐100‐5p or miR‐125b‐5p were present in 832 of the 1400 most downregulated genes (59.4%) for TCam2 cells (197 genes with the miR‐100‐5p SCR, *Sylamer*‐derived *P* = 2.71 × 10^−21^; and 793 genes with the miR‐125b‐5p SCR, *P* = 9.68 × 10^−16^; 158 overlapping) and 852 of the 1400 genes (60.9%) for 1411H cells (179 genes with the miR‐100‐5p SCR, *P* = 2.66 × 10^−9^; and 830 genes with miR‐125b‐5p SCR, *P* = 3.18 × 10^−12^; 157 overlapping) (Fig. S8A, Table S8A). At d7, the SCRs for miR‐100‐5p or miR‐125b‐5p were present in 800 of the 1400 most downregulated genes (57.1%) in TCam2 cells (164 genes with miR‐100‐5p SCR, *Sylamer*‐derived *P* = 1.01 × 10^−5^; and 776 genes with miR‐125b‐5p SCR, *P* = 1.82 × 10^−7^; 140 overlapping) and 831 of the 1400 genes (59.4%) for 1411H cells (167 genes with miR‐100‐5p SCR, *P* = 6.92 × 10^−5^; and 805 genes with miR‐125b‐5p SCR, *P* = 2.40 × 10^−8^; 141 overlapping) (Fig. S8B, Table S8A). As expected, the *Sylamer*‐derived *P*‐values for enrichment for SCRs of the miRNAs of interest were more modest at d7 compared with d2 results, as direct mRNA effects at this timepoint are less substantial and more secondary (indirect) effects occur. Further, it should be noted that the greater numbers of target genes observed for miR‐125b‐5p compared with miR‐100‐5p in these analyses were as expected, given that the background rate of occurrence of the 2–7 nt SCR sequence for miR‐125b‐5p (TCAGGG) was 31.8% (2768 of, e.g., 8706 fully annotated mRNA microarray probes) compared with 5.6% (484/8706) for miR‐99a‐5p/miR‐100‐5p (ACGGGT) (Table S8B).

### 3.7 Validation of mRNA microarray data following combination miR‐100‐5p/miR‐125b‐5p replenishment in malignant GCT cell lines

**Fig. 4:**
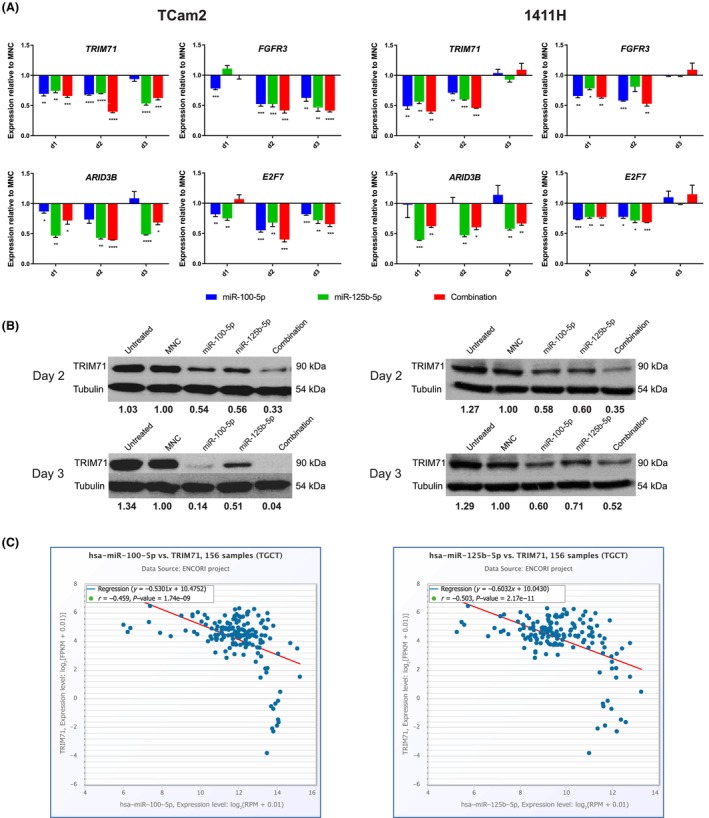
Validation of downregulated miR‐99a‐5p/miR‐100‐5p and miR‐125b‐5p messenger RNA (mRNA) targets in malignant germ cell tumor (GCT) cells following microRNA (miRNA) replenishment. (A) Quantitative RT‐PCR (qRT‐PCR) validation of expression levels in TCam2 (left) and 1411H (right) cells of the four downregulated mRNA targets *TRIM71*, *FGFR3*, *ARID3B*, and *E2F7*, that all contained the seed complementary region (SCR) for both miR‐99a‐5p/miR‐100‐5p and miR‐125b‐5p, identified from *Sylamer* analysis of microarray data at day (d) two following combination miR‐100‐5p/miR‐125b‐5p replenishment. Levels shown are for day 1 (d1), d2, and d3 post‐miRNA replenishment. For this work, cells were treated as described in Fig. 3, namely total miRNA/mimic‐negative‐control (MNC) concentrations were used at 16.7 nm; and for combination miRNA mimics, this was an 8.33 nm equimolar concentration of each. Target expression levels for these replenishment conditions are as per the color‐code below the qRT‐PCR plots. Error bars = standard error of the mean (SEM). Statistical significance (Student's *t*‐test): *P ≤ 0.05, **P < 0.01, ***P < 0.005, ****P < 0.001. Only significant comparisons are shown. Note that all experiments were performed in biological triplicate (i.e., *n* = 3) for individual treatment conditions, each with technical replicates. (B) Further validation of downregulated TRIM71 at the protein level at d2 and d3 by representative western blot for TCam2 (left) and 1411H (right) cells, from experiments performed in biological triplicate (i.e., *n* = 3). Tubulin protein is used as a loading control. The experimental condition used is shown above each blot. The quantification number under each band on the blot represents densitometry analysis of protein expression normalized to tubulin using imagej software and referenced to expression in MNC‐treated cells, which are given an arbitrary value of 1.00. The size of the specific protein is listed in kilodaltons (kDa) on the right‐hand side of the blots. (C) Co‐expression analysis of miR‐100‐5p levels (left) and miR‐125b‐5p levels (right) (*x*‐axis) with *TRIM71* mRNA levels (*y*‐axis) in malignant GCT tissue samples (*n* = 156). Data obtained from the independent ENCORI Pan‐Cancer Analysis Platform. For the co‐expression analysis in (C), statistical significance is determined using linear regression analysis; the red line indicates the best fit line based on this analysis.

We iteratively selected four miR‐99a‐5p/miR‐100‐5p and miR‐125b‐5p SCR‐containing genes for qRT‐PCR validation that were downregulated at d2 following combination miR‐100‐5p/miR‐125b‐5p replenishment from the 1400 downregulated gene lists derived from *Sylamer* analysis. We selected *TRIM71* (TCam2: rank 11 and 1411H: 220), predominantly downregulated in TCam2; *fibroblast growth factor receptor 3* (*FGFR3*) (TCam2: 20 and 1411H: 14) and *AT‐rich interaction domain 3B* (*ARID3B*) (TCam2: 53 and 1411H: 18), substantially downregulated in both; and finally, *E2F transcription factor 7* (*E2F7*) (TCam2: 368 and 1411H: 967), weakly downregulated in both, in order to validate genes across the range of downregulation observed. For this work, levels of these four genes were validated by qRT‐PCR, as expected, in TCam2 and 1411H cells at d1, d2, and d3 post‐miRNA replenishment (Fig. 4A). We further validated the findings for TRIM71 at the protein level at d2 and d3 by western blot in both TCam2 and 1411H cells (Fig. 4B). Importantly, co‐expression analysis in the large independent ENCORI/starBase Pan‐Cancer Analysis Platform dataset of 156 cases (https://rnasysu.com/encori/) [41] confirmed a very strong negative correlation between *TRIM71* mRNA levels and both miR‐100‐5p and miR‐125b‐5p levels in malignant GCT tissues (miR‐100‐5p, *R* ^2^ value = −0.459, *P* = 1.74 × 10^−9^; miR‐125b‐5p, *R* ^2^ value = −0.503, *P* = 2.17 × 10^−11^) (Fig. 4C). Similarly strong negative correlations were also observed for *FGFR3* and *ARID3B* with both miRNAs (Fig. S9A,B). Consistent with weak downregulation in both TCam2 and 1411H cell lines, *E2F7* only demonstrated a weak negative correlation in tissue samples (Fig. S9C). As each miRNA can target hundreds of mRNAs, changes in expression levels of individual mRNAs and proteins following miRNA perturbation are typically very subtle [38]. Accordingly, rather than undertaking extensive validation at the protein level, which is time‐consuming, expensive, and of limited value, such multiple subtle shifts in expression levels are best assessed by global and pathway analyses on mRNA data [22], as described in detail below.

### 3.8 Metascape pathway analyses of downregulated genes in malignant GCT cell lines at d2 and d7 following combination replenishment with miR‐100‐5p and miR‐125b‐5p

**Fig. 5:**
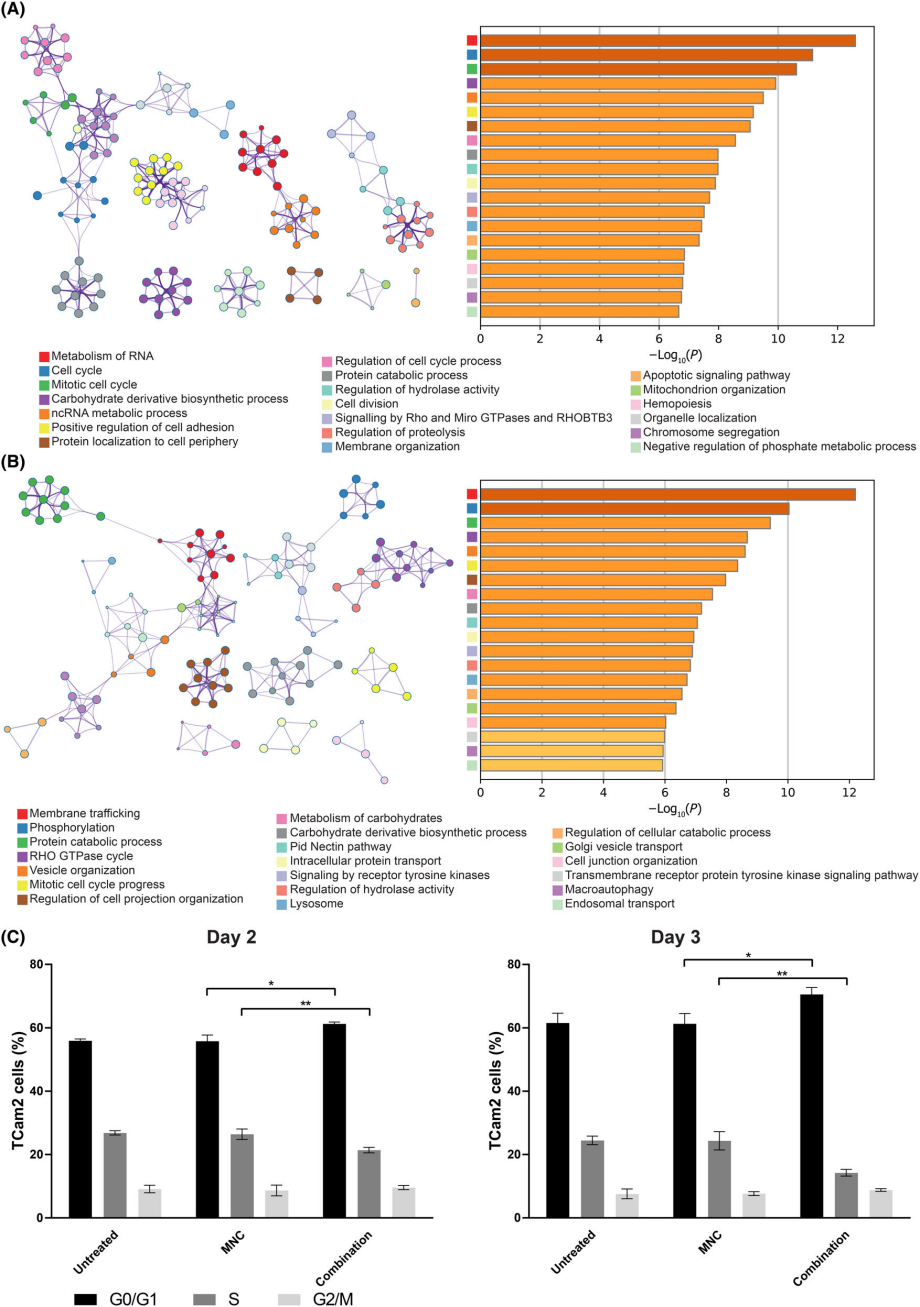
Metascape pathway analysis in malignant germ cell tumor (GCT) cells at day 2 (d2) following combination miR‐100‐5p and miR‐125b‐5p replenishment. Bar plots (right) and associated network analyses (left) for (A) TCam2 (d2; 832 genes) and (B) 1411H (d2; 852 genes), showing the top‐20 most significant functional pathways for the downregulated seed complementary region (SCR)‐containing messenger RNA (mRNA) targets of miR‐100‐5p or miR‐125b‐5p following 16.7 nm combination microRNA (miRNA) replenishment. (C) Bar plots of the percentage (%) of TCam2 cells in G0/G1‐ (black), S‐ (dark gray) and G2/M‐ (light gray) phase of the cell cycle as assessed by flow cytometry at d2 and day 3 (d3) following 16.7 nm combination miR‐100‐5p and miR‐125b‐5p replenishment, compared with mimic‐negative‐control (MNC)‐treated and untreated cells. Data represent the mean and error bars = standard error ofthe mean (SEM) of biological triplicate values. Statistical significance (Student's *t*‐test) with the appropriate MNC comparison: *P ≤ 0.05, **P < 0.01. Only significant comparisons are shown.

The top‐20 most significant functional pathways for the 832 and 852 downregulated SCR‐containing mRNA targets of miR‐100‐5p or miR‐125b‐5p in TCam2 and 1411H cells, respectively, at d2 following combination miRNA replenishment are shown as enrichment analyses (Tables S9 and S10), bar plots, and associated network analyses (Fig. 5A,B). The most significant pathways for TCam2 included cell cycle, cell division, and chromosome segregation; cell adhesion; and GTPase signaling (Fig. 5A). Likewise, for 1411H, key pathways included cell cycle; GTPase signaling; and endosomal transport and vesicle organization (Fig. 5B). The top‐20 most significant functional pathways for the 800 and 831 downregulated SCR‐containing mRNA targets of miR‐100‐5p or miR‐125b‐5p in TCam2 and 1411H cells, respectively, at d7 following combination miRNA replenishment are shown as enrichment analyses (Tables S11 and S12), bar plots, and associated network analyses (Fig. S10). Significant pathways in TCam2 included retention of cell cycle and GTPase‐associated pathways, which comprised five of the top‐20 significant terms, and for 1411H the cell cycle term was also retained (Fig. S10). The full lists of downregulated genes following combination miRNA replenishment in TCam2 and 1411H cells at d2 and d7 and target information are detailed in Tables [Link], [Link], [Link], [Link], respectively. Consistent with the pathway analysis described, cell cycle analysis demonstrated an increased proportion of malignant GCT cells in G0/G1‐phase and a concomitant reduced proportion in S‐phase at d2 and d3 following combination miR‐100‐5p/miR‐125b‐5p replenishment (Fig. 5C). Next, we undertook Metascape analysis on the 734 unique upregulated miR‐100‐5p or miR‐125b‐5p mRNA targets from clinical malignant GCT tissue samples, shown as a bar plot (Fig. S11). Importantly, within the top‐20 significant pathways, GTPase signaling was included twice, overlapping with both TCam2 and 1411H analyses, in addition to cell adhesion. Together, these data indicate that our findings in malignant GCT cell lines hold clinical relevance.

### 3.9 Knockdown of *TRIM71* (*TRIM71*kd), a miR‐100‐5p and miR‐125b‐5p target, recapitulates combination miRNA replenishment through cell cycle disruption

**Fig. 6:**
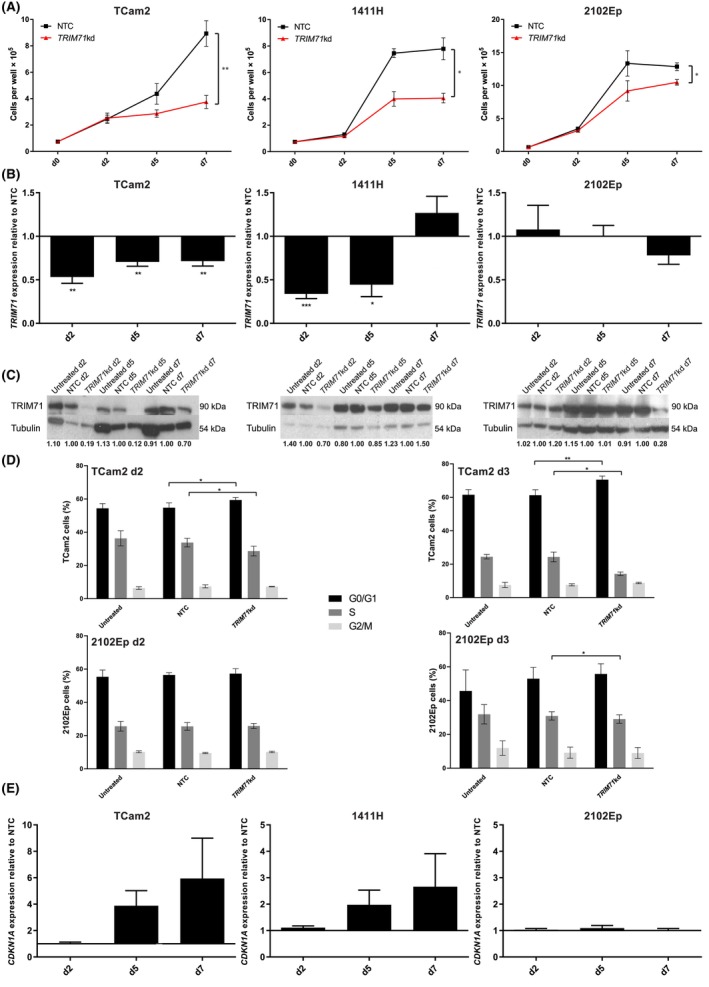
Knockdown of *TRIM71* (*TRIM71*kd) recapitulates combination miR‐100‐5p and miR‐125b‐5p replenishment in malignant germ cell tumor (GCT) cells. (A) Growth curves for TCam2 (left), 1411H (center), and 2102Ep (right) cells following transfection with 40 nm *TRIM71* or nontargeting control (NTC) short interfering RNA (siRNA) compared with untreated cells. (B) *TRIM71* messenger RNA (mRNA) levels by quantitative RT‐PCR (qRT‐PCR) and (C) representative TRIM71 protein levels by western blotting for malignant GCT cells corresponding to the growth curves in (A), from experiments performed in biological triplicate (i.e., *n* = 3). Tubulin protein is used as a loading control. The experimental condition used is shown above each blot. The quantification number under each band on the blot represents densitometry analysis of protein expression normalized to tubulin using imagej software and referenced to expression in nontargeting control (NTC)‐treated cells, which are given an arbitrary value of 1.00. The size of the specific protein is listed in kilodaltons (kDa) on the right‐hand side of the blots. (D) Bar plots of the percentage (%) of malignant GCT cells for TCam2 (upper panels) and 2012Ep (lower panels) in G0/G1‐ (black), S‐ (dark gray), and G2/M‐ (light gray) phase of the cell cycle as assessed by flow cytometry at day 2 (d2) and d3 following 40 nm *TRIM71*kd, compared with NTC‐treated and untreated cells. (E) *CDKN1A* mRNA levels by qRT‐PCR in malignant GCT cells following 40 nm *TRIM71*kd. Note that all experiments in (A, B, D, E) were performed in biological triplicate (i.e., *n* = 3) for individual treatment conditions, each with technical replicates; the data shown in (A, B, D, E) represent the mean and error bars = standard error of the mean (SEM) of these biological triplicate values. Statistical significance (Student's *t*‐test) with the appropriate NTC comparison: *P ≤ 0.05, **P < 0.01, ***P < 0.005. Only significant comparisons are shown.

Having identified *TRIM71* as a key downregulated miR‐100‐5p/miR‐125b‐5p target in malignant GCT cell lines (Fig. 4A,B) and a strong negative correlation of *TRIM71* with miR‐100‐5p/miR‐125b‐5p levels in malignant GCT tissues (Fig. 4C), we next sought to confirm this as a direct target using a luciferase reporter assay. However, no *TRIM71* reporter with a full‐length 3′UTR (6013 kb) is commercially available, and, furthermore, the very long 3′UTR prevented successful cloning to allow creation of our own *TRIM71* reporter, despite multiple attempts. Instead, we explored the functional significance of these highly significant correlations with *TRIM71* through knockdown (kd) experiments. Importantly, *TRIM71* promotes G1‐S transition to allow rapid embryonic stem cell (ESC) division, with *TRIM71*kd prolonging the G1 phase of the cell cycle [42]. Similarly, *TRIM71*kd growth curves in TCam2, 1411H, and 2102Ep malignant GCT cells resulted in significant growth inhibition at d7 compared with cells transfected with nontargeting control (NTC) siRNA (*P* ≤ 0.05 for each cell line) (Fig. 6A). This was associated with maximal reductions at the *TRIM71* mRNA level by 46.8%, 66.2%, and 22.0%, respectively (Fig. 6B), findings confirmed by western blotting analysis (Fig. 6C). Of note, different *TRIM71* kinetics were observed, with maximal mRNA and protein knockdown at d2 and d5, respectively, for TCam2, d2 for both for 1411H, and d7 for both for 2102Ep (Fig. 6B,C). Overall, cell cycle analysis at d2 and d3 demonstrated increased proportions of malignant GCT cells in G0/G1‐phase and a concomitant reduced proportion in S‐phase following *TRIM71*kd (Fig. 6D). Finally, in malignant GCT cells displaying earlier (d2/d5) *TRIM71*kd, namely TCam2 and 1411H, increases in transcript levels of *cyclin‐dependent kinase inhibitor 1A* (*CDKN1A*, which encodes the p21 protein) were observed (Fig. 6E), as expected given the role of TRIM71 as an mRNA repressor of *CDKN1A*, resulting in degradation [43].

### 3.10 Involvement of miR‐125b‐5p in the *lin‐28 homolog A* (*LIN28A*)‐*let‐7* feedback loop in malignant GCTs

**Fig. 7:**
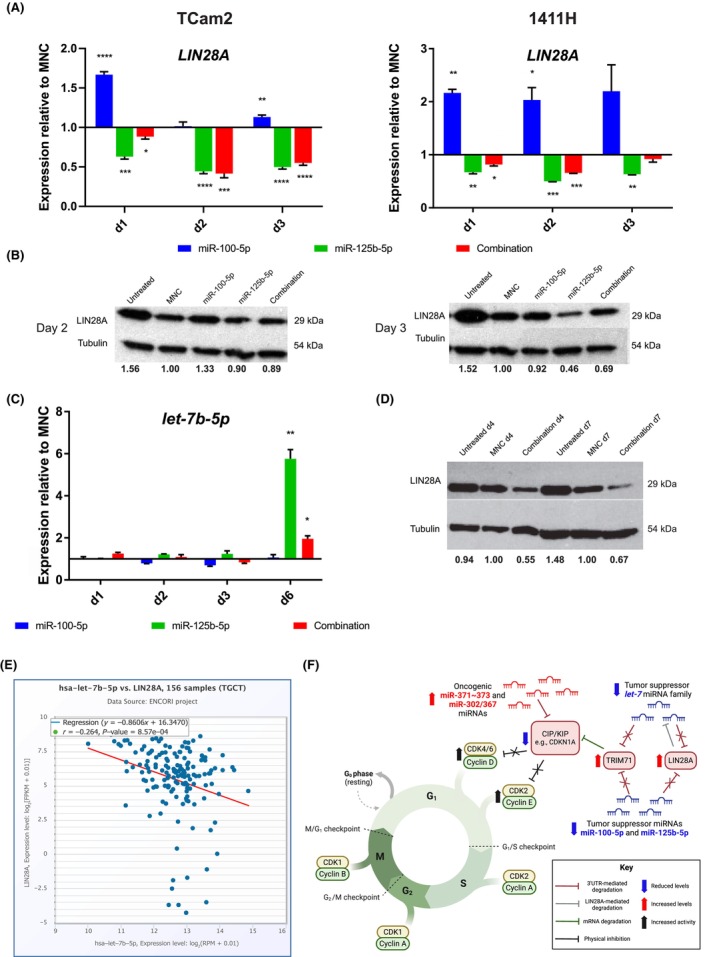
Combination miR‐100‐5p/miR‐125b‐5p replenishment results in *LIN28A* depletion and late concomitant increases in *let‐7* tumor suppressor microRNA (miRNA) levels in malignant germ cell tumor (GCT) cells. (A) *LIN28A* quantitative RT‐PCR (qRT‐PCR) levels in TCam2 (left) and 1411H (right) cells at day (d) 1, d2, and d3 following microRNA (miRNA) replenishment. For this work, cells were treated as described in Fig. 3, namely total miRNA/mimic‐negative‐control (MNC) concentrations were used at 16.7 nm; and for combination miRNA mimics, this was an 8.33 nm equimolar concentration of each, as per the color‐code below the qRT‐PCR plots. (B) Representative LIN28A protein levels by western blot on d2 and d3 in TCam2 cells. (C) *Let‐7b‐5p* miRNA expression levels as quantified by qRT‐PCR at d1, d2, d3, and d6, and (D) representative LIN28A protein levels by western blot at d4 and d7; both following mimic replenishment in TCam2 cells as detailed in (A) above. Note that the experiments in (A) and (C) were performed in biological triplicate (i.e., *n* = 3) for individual treatment conditions, each with technical replicates; the data shown in (A) and (C) represent the mean and error bars = SEM of these biological triplicate values. For (A) and (C), statistical significance using Student's *t*‐test is shown with the appropriate comparisons: *P ≤ 0.05, **P < 0.01, ***P < 0.005, ****P < 0.001. Only significant comparisons are shown. For representative western blots shown in (B) and (D), these were from experiments performed in biological triplicate (i.e., *n* = 3), with tubulin protein used as a loading control. The experimental condition used is shown above each blot. The quantification number under each band on the blot represents densitometry analysis of protein expression normalized to tubulin using imagej software and referenced to expression in mimic‐negative‐control (MNC)‐treated cells, which are given an arbitrary value of 1.00. The size of the specific protein is listed in kilodaltons (kDa) on the right‐hand side of the blots. (E) Co‐expression analysis of *let‐7b‐5p* levels (*x*‐axis) with *LIN28A* mRNA levels (*y*‐axis) in malignant GCT tissue samples (*n* = 156). Data obtained from the independent ENCORI Pan‐Cancer Analysis Platform. For the co‐expression analysis in (E), statistical significance is determined using linear regression analysis; the red line indicates the best fit line based on this analysis. (F) Graphical abstract summarizing potential miRNA effects on the cell cycle in malignant GCTs derived from data from this manuscript and published studies. Reduced levels (blue arrows) of tumor suppressor miR‐100‐5p/miR‐125b‐5p miRNAs result in increased levels (red arrows) of, for example, *TRIM71*, with concomitant reductions in cyclin‐dependent kinase (CDK)‐interacting protein/kinase inhibitory protein (CIP/KIP) family members such as *CDKN1A* (blue arrow) [43], coding for p21 protein. In turn, this allows cell cycle progression through reduced physical inhibition (i.e., increased activity; black arrows) of CDK2 and CDK4 [58]. As demonstrated in this study, replenishment of these downregulated miRNAs, or *TRIM71*kd, reduces cell cycle proliferation through converse effects on the pathway and thus G0/G1 and G1/S transition. Further contributions to malignant GCT pathogenesis are likely from, for example, reduced miRNA‐mediated control of *LIN28A* levels. High *LIN28A* levels cause increased degradation of *let‐7* tumor suppressor miRNA family members, reducing its own inhibition via the *LIN28A*/*let‐7* feedback loop, resulting in cellular proliferation [21]. To add to the complexity, *TRIM71* itself is also a direct mRNA target of the *let‐7* family [42]. Finally, the effects of overexpressed oncogenic miR‐371~373 and miR‐302/367 miRNAs also converge on the cell cycle through CIP/KIP family members [22]. 3′UTR, 3′ untranslated region. Schematic created with BioRender.com.

We previously demonstrated the importance of the *LIN28A*‐*let‐7* feedback loop in malignant GCTs [21] and subsequently hypothesized that the high *LIN28A* levels observed in these tumors may be due to the downregulation of other miRNAs which are predicted to target *LIN28A*, such as miR‐125b‐5p [3]. Importantly, in this study we identified *LIN28A* as one of the most downregulated genes following combination miR‐100‐5p/miR‐125b‐5p replenishment (TCam2 d2: rank 1, TCam2 d7: rank 2, and 1411H d7: rank 6). Further, we noted that the 3′ untranslated region (3′UTR) of *LIN28A* had five miR‐125b‐5p binding sites (and none for miR‐100‐5p). To confirm the direct specificity of this finding, we studied *LIN28A* qRT‐PCR levels in TCam2 and 1411H cells at d1, d2, and d3 post‐miRNA replenishment with miR‐100‐5p 16.7 nm alone, miR‐125b‐5p 16.7 nm alone, and combination miR‐100‐5p/miR‐125b‐5p 16.7 nm replenishment (i.e., an equimolar 8.33 nm concentration of each) (Fig. 7A). This confirmed, as expected, significant downregulation of *LIN28A* in both cell lines at all timepoints for miR‐125b‐5p replenishment, with more modest downregulation for the combination treatment (where the miR‐125b‐5p concentration was only 8.33 nm), particularly for 1411H (where significant *LIN28A* downregulation was not observed until d7 in the microarray data) (Fig. 7A). These findings are consistent with the *Sylamer* gene list rankings. Next, we confirmed these observations at the protein level by western blot on d2 and d3 in TCam2 (Fig. 7B). Consistent with previous work, where levels of *let‐7* family members started increasing from d3 following LIN28A protein depletion [21], we observed *let‐7* upregulation (*MIRLET7B/let‐7b‐5p*) in TCam2 cells at d6 after original miR‐125b‐5p replenishment (Fig. 7C), and 3–4 days following the confirmed reduction of LIN28A protein levels at d2/3 (Fig. 7B). In turn, these late *let‐7* increases resulted in sustained depletion of LIN28A protein levels following original miR‐125b‐5p replenishment, as highlighted by persistently low levels at d7, in addition to d4 (Fig. 7D). Importantly, the clinical relevance of these observations was also confirmed by co‐expression analysis in the large ENCORI dataset [41], showing a strong negative correlation between *LIN28A* mRNA and *let‐7b‐5p* levels in malignant GCT tissues (*R* ^2^ value = −0.264, *P* = 8.57 × 10^−4^; Fig. 7E).

## 4 Discussion

Here, we have provided further evidence demonstrating a critical role for miRNA dysregulation in the pathogenesis of malignant GCTs [19]. Data demonstrating intermediate miRNA expression in the malignant GCT precursor lesion germ cell neoplasia *in situ* (GCNIS), compared with gonadal control and malignant GCT samples, provides further support for this hypothesis [44]. We first demonstrated universal downregulation of miR‐99a‐5p, miR‐100‐5p, and miR‐125b‐5p in malignant GCT clinical samples, including all individual subtypes, and cell lines, supporting our subsequent investigation of their regulation and function. A previous report has identified that further downregulation of these specific microRNAs is associated with the acquisition of cisplatin resistance in malignant GCTs [45], lending additional credence to our approach. Accordingly, we next showed that the reduced expression of mature miR‐99a‐5p/miR‐100‐5p and miR‐125b‐5p, their host lincRNAs *MIR99AHG* and *MIR100HG*, the protein‐coding gene *BLID* [46], and their primary transcript pre‐miRNAs from the corresponding chr11 and chr21 genomic loci are all strongly correlated, both within and across loci. These results suggested that these miRNAs originating from chr11 and chr 21 are transcribed as a single polycistronic sequence with their host lincRNAs, and that their transcription is regulated by a common regulatory mechanism in malignant GCTs, not specific to miRNA biogenesis. Further evidence in support of this theory is the observation that somatic mutations of *DICER1*, a critical miRNA biogenesis enzyme, occur only rarely in GCTs, with only one of 14 GCT cases harboring a mutation described in one report [47] and just 3.1% in another much larger study of 161 GCTs [48].

Approximately half of human miRNAs are closely associated with CpG islands [49], where DNA methylation occurs [50]. As methylation changes are highly dynamic during germ cell development and hypermethylation of tumor suppressor genes has been reported in malignant GCT pathogenesis [51], we next sought to identify whether this mechanism had a role in the miR‐99a‐5p/miR‐100‐5p and miR‐125b‐5p downregulation observed in malignant GCTs, having established that there was no evidence of genomic copy number loss to account for this downregulation. Treatment of malignant GCT cells using the demethylating agent 5‐azacytidine confirmed concomitant increased levels of miR‐99a‐5p/miR‐100‐5p and miR‐125b‐5p, as well as *MIR100HG*, *MIR99AHG*, and *BLID*. Subsequent pyrosequencing studies generally confirmed hypermethylation upstream of the chr11 and chr21 loci coding for these miRNAs in malignant GCT cell lines, consistent with other reports showing miR‐100 was downregulated due to hypermethylation of the host gene *MIR100HG* [15], and silencing of miR‐125b‐5p was due to hypermethylation, including in GCTs [16, 52]. Some differences in methylation were observed between malignant GCT subtypes, as expected, given they originate from germ cells at different developmental stages [53], with NSGCTs being more hypermethylated than Sem [54, 55, 56], consistent with our analyses of published data for both malignant cell lines and tissues [34]. Taken together, we demonstrated that upstream hypermethylation of the chr11 and chr21 loci in malignant GCT cells is likely to contribute to the observed downregulation of miRNAs miR‐99a‐5p/miR‐100‐5p, miR‐125b‐5p, and their associated genes.

Next, we investigated the functional significance of miR‐99a‐5p/miR‐100‐5p and miR‐125b‐5p downregulation in malignant GCTs. Rather than use the 5‐azacytidine proof‐of‐principle studies to examine functional effects, which risked off‐target or nonspecific effects mediated via other genes and pathways, we studied direct replenishment of the miRNAs *per se*. Given miR‐99a‐5p and miR‐100‐5p have an identical 2‐7 nt seed sequence and differ by only a single nucleotide at the 3′ end, and miR‐99a‐5p/miR‐100‐5p and miR‐125b‐5p have coregulated expression, we delivered combination replenishment of miR‐100‐5p and miR‐125b‐5p and showed growth inhibition in malignant GCT cell lines, specifically TCam2 (Sem) and 1411H (YST). This phenotypic effect was associated with global genotypic effects in these cell lines, with highly significant enrichment for miR‐99a‐5p/miR‐100‐5p and/or miR‐125b‐5p targets in both downregulated gene lists using *Sylamer* analysis.

Metascape pathway analysis revealed consistent cell cycle effects following combination miRNA replenishment in both TCam2 and 1411H cell lines on d2 analyses, which importantly persisted through to d7. Of note, this cell cycle disruption is consistent with observations when targeting overexpressed miRNAs in malignant GCTs [22]. Flow cytometry experimentally confirmed the effects on the cell cycle. Other important pathways identified likely cancer‐associated terms, including GTPase signaling, cell adhesion, and endosomal transport and vesicle organization.

Selected *Sylamer*‐derived targets from within the downregulated gene lists were validated, including *TRIM71*, *ARID3B*, *and FGFR3*, the latter a direct target of miR‐100 in osteosarcoma [57]. Consistent with our pathway analysis observations, *TRIM71* knockdown (*TRIM71*kd) prolongs G1 phase and slows cell proliferation in ESCs [42], also shown here for malignant GCTs. TRIM71 effects are mediated through *CDKN1A* [43], which codes for the p21 protein, which in turn regulates downstream cyclin‐dependent kinases (CDKs) via physical interaction/inhibition rather than altered expression levels [58], summarized graphically in Fig. 7F. Similarly, *ARID3B* knockdown also attenuates cell cycle progression and suppresses tumor growth [59]. The findings for *FGFR3* are of particular interest, with analysis of publicly available The Cancer Genome Atlas (TCGA; https://www.cancer.gov/ccg/research/genome‐sequencing/tcga) data, and comparison with Genotype‐Tissue Expression (GTEx; https://gtexportal.org/home/) normal control tissues, also confirming upregulated *FGFR3* expression in malignant GCTs [60]. Of clinical relevance, the *FGFR3* pathway is already amenable to novel therapeutic inhibition with existing therapies. While sensitivity to most FGFR‐selective tyrosine kinase inhibitors such as lenvatinib, ponatinib, regorafenib, and erdafitinib requires specific hotspot mutations, other agents targeting FGFR3 are more permissive and have activity in mutation‐negative models. For example, rogaratinib is a selective pan‐FGFR kinase inhibitor (including FGFR3) with broad antitumor activity in FGFR‐overexpressing preclinical cancer models, mediated through FGFR/ERK (*mitogen‐activated protein kinase 1*) pathway inhibition and resulting in reduced cell proliferation [61]. Further, rogaratinib demonstrated strong *in vivo* efficacy in several FGFR‐overexpressing models including patient‐derived xenografts, with efficacy strongly correlated with *FGFR* mRNA expression levels [61]. Another agent of interest is vofatamab (B‐701), a selective human monoclonal antibody against FGFR3 that blocks activation of both the wild‐type and genetically activated receptor, and which, like rogaratinib, is in early‐phase clinical trials.

We previously demonstrated the importance of the *LIN28A*‐*let‐7* feedback loop in malignant GCTs, demonstrating a strong negative correlation between *LIN28A* and *let‐7* levels in matched clinical specimens [21]. Further, *LIN28A* knockdown or *let‐7* miRNA replenishment resulted in reduced proliferation in malignant GCT cells, across the range of commonly observed histological subtypes, with *LIN28A* confirmed as a direct *let‐7* target in this setting, highlighting the functional significance of this correlation [21]. We also hypothesized that high *LIN28A* levels observed may be due to the downregulation of other miRNAs which are predicted to target *LIN28A*, such as miR‐125b‐5p [3]. Here, we also experimentally confirmed that miR‐125b‐5p replenishment resulted in prolonged downregulation of *LIN28A* levels with corresponding late increases in *let‐7* miRNAs at d6, as expected. To add to the complexity, *TRIM71* itself is also a direct mRNA target of *let‐7* [42]. Together, these data highlight the multilayer complexity of miRNA dysregulation in malignant GCTs and highlight that miRNA replenishment offers both shorter‐ and longer‐term effects resulting in sustained reversion of cell phenotype (Fig. 7F). Finally, analysis of global gene expression profiling data from malignant GCT cell lines treated with combination miRNA replenishment (downregulated direct targets) with clinical tissue samples (upregulated direct targets) revealed overlap of affected pathways, demonstrating the clinical relevance of our *in vitro* studies.

We acknowledge that further work is now necessary and warranted in malignant GCTs, focusing on additional *in vitro* experimentation such as soft agar anchorage‐independent growth assays, further attempted targeting of EC cell lines, and *in vivo* models, for example, xenograft studies in immunocompromised mice, in order to directly explore the role of miR‐100‐5p and miR‐125b‐5p in other hallmarks of tumorigenicity in addition to cell cycle disruption. It will also be important to use miRNA replenishment with platinum agents to study potential synergy which may result in effective future treatment using lower conventional chemotherapy doses, outside the remit of the current study.

## 5 Conclusion

In summary, these findings further our understanding of the mechanisms and functions of downregulated miRNAs in malignant GCTs. This study identified a possible regulatory mechanism likely to contribute to miR‐99a‐5p/miR‐100‐5p and miR‐125b‐5p downregulation in this tumor type and that combined replenishment resulted in sustained phenotypic and genotypic changes *in vitro*. The clinical potential for replenishment strategies for downregulated miRNAs in malignant GCTs has been highlighted [19], and more widely via a recent clinical trial of miRNA replenishment therapy in cancer [20]. This work provides an essential platform for the additional *in vitro* and *in vivo* studies that are now warranted to explore the potential of miRNA replenishment therapy and/or existing available novel agents for affected pathways such as *FGFR3* in malignant GCTs, with the aim of improving overall and quality of survival for patients affected by this disease.

## Conflict of interest

The authors declare no conflict of interest.

## Author contributions

CGS, NC, and MJM were involved in study conceptualization. MF, SB, LA‐C, DW, CP, HKS, SPS, AJE, CGS, and MJM were involved in obtaining experimental data. MF, SB, LA‐C, DW, CP, ZGLS, HKS, SPS, JCN, AJE, CGS, NC, and MJM were involved in data curation, interpretation, and analysis. CGS, NC, and MJM were involved in supervision. MF, CGS, NC, and MJM were involved in writing—original manuscript draft. MF, SB, LA‐C, DW, CP, ZGLS, HKS, SPS, JCN, AJE, CGS, NC, and MJM were involved in writing—review and editing, approval of final manuscript.

## Supporting information


**Fig. S1.** Confirmatory quantitative RT‐PCR (qRT‐PCR) data showing relative miR‐99a‐5p/miR‐100‐5p and miR‐125b‐5p expression in malignant germ cell tumor (GCT) clinical samples (*n* = 24) and cell lines (*n* = 7) compared with controls (*n* = 2).
**Fig. S2.** Genomic loci and expression levels of genes of interest on chromosomes 11 and 21 in malignant germ cell tumors (GCTs).
**Fig. S3.** Genomic copy number data across the regions of interest on chromosomes 11 and 21 in malignant germ cell tumors (GCTs).
**Fig. S4.** Overall evidence that hypermethylation at chromosome 11 and 21 microRNA (miRNA) loci contributes to miR‐99a‐5p/miR‐100‐5p, miR‐125b‐5p, and related long interspersed non‐coding RNA (lincRNA) and protein‐coding gene downregulation in malignant germ cell tumors (GCTs) and cell lines.
**Fig. S5.** Lack of phenotypic effects of combination miR‐100‐5p and miR‐125b‐5p replenishment in 2102Ep (embryonal carcinoma) malignant germ cell tumor (GCT) cells and potential explanation through enhanced excretion in extracellular vesicles (EVs).
**Fig. S6.** Using the change‐point detection algorithm to determine the optimal peaks in the Sylamer landscape plots at day 2 (d2) following 16.7 nm combination miR‐100‐5p/miR‐125b‐5p replenishment.
**Fig. S7.** *Sylamer* assessment for persistent seed complementary region (SCR) enrichment in downregulated genes at day 7 (d7) following combination miR‐100‐5p/miR‐125b‐5p replenishment at 16.7 nm.
**Fig. S8.** Schematic showing the number of downregulated miR‐99a‐5p/miR‐100‐5p or miR‐125b‐5p mRNA target genes in malignant germ cell tumor (GCT) cell lines following combination miR‐100‐5p/miR‐125b‐5p replenishment at 16.7 nm.
**Fig. S9.** Negative correlation between miR‐100‐5p and miR‐125b‐5p levels versus *FGFR3*, *ARID3B*, and *E2F7* mRNA levels in malignant germ cell tumor (GCT) tissue samples.
**Fig. S10.** Metascape pathway analysis in malignant germ cell tumour (GCT) cells at day 7 (d7) following combination miR‐100‐5p and miR‐125b‐5p replenishment at 16.7 nm.
**Fig. S11.** Metascape pathway analysis on the 734 unique upregulated miR‐100‐5p or miR‐125b‐5p messenger RNA (mRNA) targets from clinical malignant germ cell tumor (GCT) tissue samples.

**Table S1.** Clinico‐pathological data for the germ cell tumor (GCT) pediatric clinical samples and GCT cell lines used in the study.
**Table S2.** Primers used for genomic DNA (gDNA) copy number assessment across the regions of interest on chromosomes 11 and 21 in malignant germ cell tumor (GCT) cell lines and normal testis.
**Table S3.** Initial polymerase chain reaction (PCR) screening across the upstream regions of interest of the genes on (A) chromosome 11 (11q24.1) and (B) chromosome 21 (21q21.1).
**Table S4.** List of primers used for formal methylation assessment of the regions of interest on (A) chromosome 11 (11q24.1) and (B) chromosome 21 (21q21.1).
**Table S5.** Illumina messenger RNA (mRNA) microarray information.
**Table S6.** Elements comprising the seed complementary regions (SCRs) for the microRNA (miRNA) seed regions of interest for derivation of the *Sylamer* single summed significance score (SSSS).
**Table S7.** Downregulation of miR‐99a‐5p, miR‐100‐5p, and miR‐125b‐5p in malignant germ cell tumors (GCTs), GCT subtypes, and cell lines relative to non‐malignant (gonadal and teratoma) control samples; derived from global microRNA (miRNA) microarray expression data.
**Table S8.** Enrichment for seed complementary regions (SCRs) corresponding to the seed regions of miR‐99a‐5p/miR‐100‐5p and miR‐125b‐5p in 3′ untranslated regions (3′UTRs) of messenger RNA (mRNA) data.
**Table S9.** The top‐20 deregulated Metascape pathways in TCam2 cells at day 2 (d2) after combination miR‐100‐5p/miR‐125b‐5p replenishment.
**Table S10.** The top‐20 deregulated Metascape pathways in 1411H cells at day 2 (d2) after combination miR‐100‐5p/miR‐125b‐5p replenishment.
**Table S11.** The top‐20 deregulated Metascape pathways in TCam2 cells at day 7 (d7) after combination miR‐100‐5p/miR‐125b‐5p replenishment.
**Table S12.** The top‐20 deregulated Metascape pathways in 1411H cells at day 7 (d7) after combination miR‐100‐5p/miR‐125b‐5p replenishment.

**Table S13.** The list of 832 downregulated genes following combination 16.7 nm miR‐100‐5p/miR‐125b‐5p replenishment of TCam2 (seminoma) malignant germ cell tumor (GCT) cells at day 2 (d2) following transfection which were either miR‐100‐5p targets (*n* = 197), miR‐125b‐5p targets (*n* = 793), or both (*n* = 158).

**Table S14.** The list of 852 downregulated genes following combination 16.7 nm miR‐100‐5p/miR‐125b‐5p replenishment of 1411H (yolk sac tumor) malignant germ cell tumor (GCT) cells at day 2 (d2) following transfection which were either miR‐100‐5p targets (*n* = 179), miR‐125b‐5p targets (*n* = 830), or both (*n* = 157).

**Table S15.** The list of 800 downregulated genes following combination 16.7 nm miR‐100‐5p/miR‐125b‐5p replenishment of TCam2 (seminoma) malignant germ cell tumor (GCT) cells at day 7 (d7) following transfection which were either miR‐100‐5p targets (*n* = 164), miR‐125b‐5p targets (*n* = 776), or both (*n* = 140).

**Table S16.** The list of 831 downregulated genes following combination 16.7 nm miR‐100‐5p/miR‐125b‐5p replenishment of 1411H (yolk sac tumor) malignant germ cell tumor (GCT) cells at day 7 (d7) following transfection which were either miR‐100‐5p targets (*n* = 167), miR‐125b‐5p targets (*n* = 805), or both (*n* = 141).

## Data Availability

Raw microarray data from cell line analyses are deposited at the EBI Array Express (https://www.ebi.ac.uk/biostudies/arrayexpress), accession no. E‐MTAB‐13464. Tissue microarray data are publicly available at Gene Expression Omnibus, accession no. GSE18155. In addition to the data deposited above, other data that support the findings of this study are available on request.
