# Supplementary material for: Replenishing co‐downregulated miR‐100‐5p and miR‐125b‐5p in malignant germ cell tumors causes growth inhibition through cell cycle disruption
Source: Mol Oncol. 2024 Nov 10;19(4):1203–28. doi: 10.1002/1878-0261.13757 (PMC11977657; doi:10.1002/1878-0261.13757)
Supplement: Supplementary file 1 — Fig. S1. Confirmatory quantitative RT‐PCR (qRT‐PCR) data showing relative miR‐99a‐5p/miR‐100‐5p and miR‐125b‐5p expression in malignant germ cell tumor (GCT) clinical samples (n = 24) and cell lines (n = 7) compared with controls (n = 2). Fig. S2. Genomic loci and expression levels of genes of interest on chromosomes 11 and 21 in malignant germ cell tumors (GCTs). Fig. S3. Genomic copy number data across the regions of interest on chromosomes 11 and 21 in malignant germ cell tumors (GCTs). Fig. S4. Overall evidence that hypermethylation at chromosome 11 and 21 microRNA (miRNA) loci contributes to miR‐99a‐5p/miR‐100‐5p, miR‐125b‐5p, and related long interspersed non‐coding RNA (lincRNA) and protein‐coding gene downregulation in malignant germ cell tumors (GCTs) and cell lines. Fig. S5. Lack of phenotypic effects of combination miR‐100‐5p and miR‐125b‐5p replenishment in 2102Ep (embryonal carcinoma) malignant germ cell tumor (GCT) cells and potential explanation through enhanced excretion in extracellular vesicles (EVs). Fig. S6. Using the change‐point detection algorithm to determine the optimal peaks in the Sylamer landscape plots at day 2 (d2) following 16.7 nm combination miR‐100‐5p/miR‐125b‐5p replenishment. Fig. S7. Sylamer assessment for persistent seed complementary region (SCR) enrichment in downregulated genes at day 7 (d7) following combination miR‐100‐5p/miR‐125b‐5p replenishment at 16.7 nm. Fig. S8. Schematic showing the number of downregulated miR‐99a‐5p/miR‐100‐5p or miR‐125b‐5p mRNA target genes in malignant germ cell tumor (GCT) cell lines following combination miR‐100‐5p/miR‐125b‐5p replenishment at 16.7 nm. Fig. S9. Negative correlation between miR‐100‐5p and miR‐125b‐5p levels versus FGFR3, ARID3B, and E2F7 mRNA levels in malignant germ cell tumor (GCT) tissue samples. Fig. S10. Metascape pathway analysis in malignant germ cell tumour (GCT) cells at day 7 (d7) following combination miR‐100‐5p and miR‐125b‐5p replenishment at 16.7 nm. Fig. S11. Meta [file MOL2-19-1203-s004.pptx]

## Slide 1
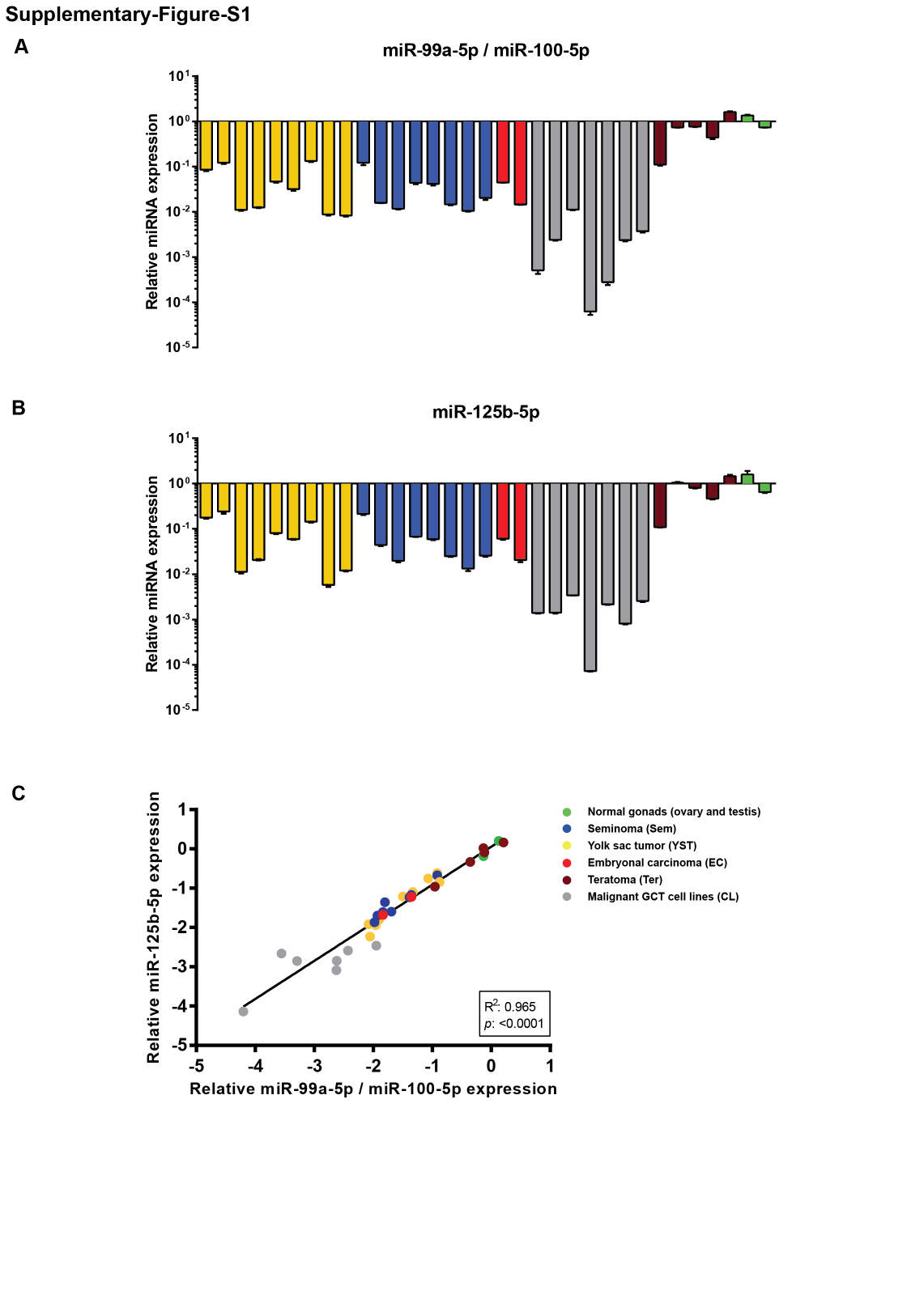

## Slide 2
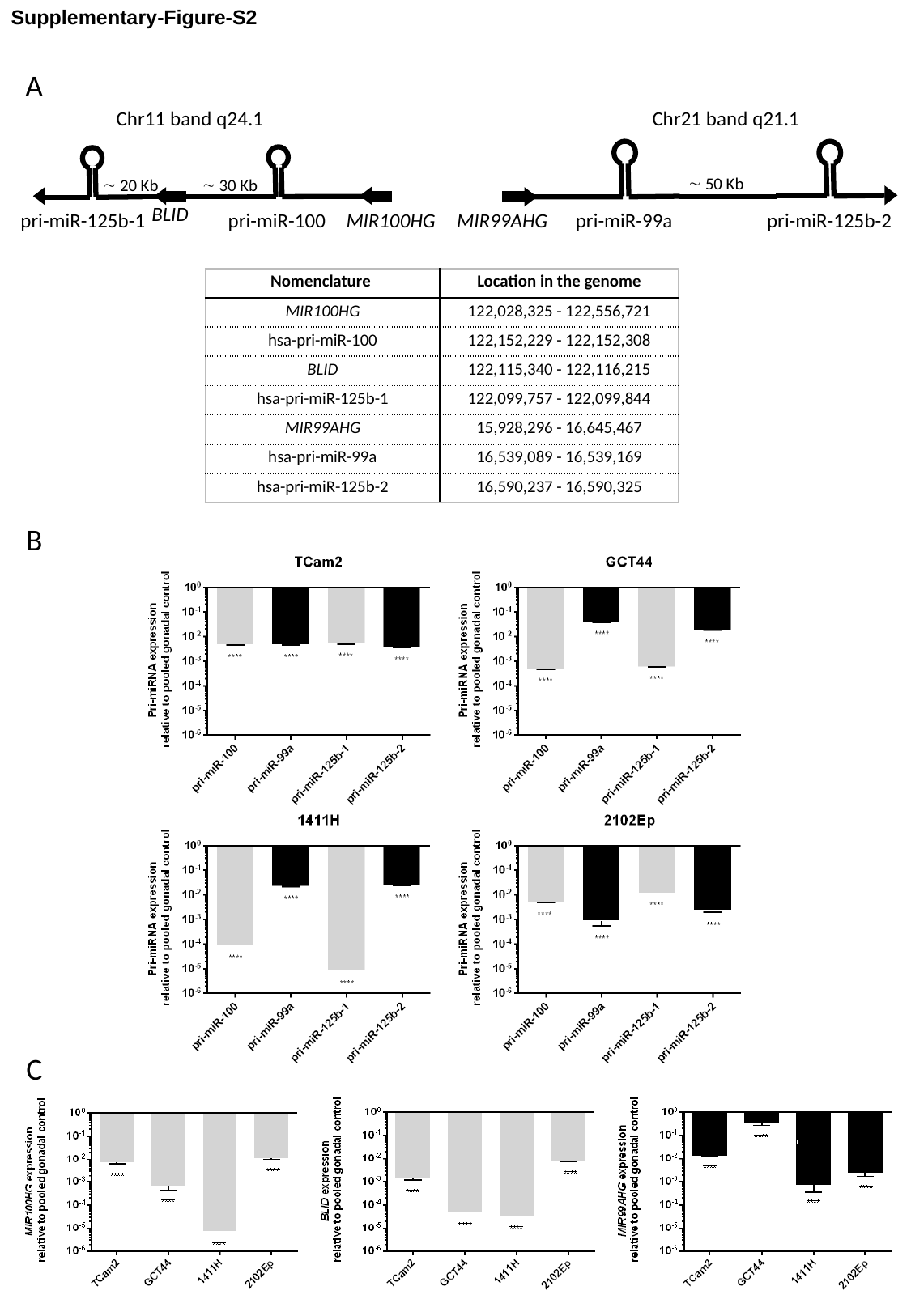

Supplementary-Figure-S2
A
Chr11 band q24.1
Chr21 band q21.1
 50 Kb
MIR99AHG
pri-miR-125b-2
pri-miR-99a
 20 Kb
 30 Kb
BLID
pri-miR-125b-1
pri-miR-100
MIR100HG
| Nomenclature | Location in the genome |
| --- | --- |
| MIR100HG | 122,028,325 - 122,556,721 |
| hsa-pri-miR-100 | 122,152,229 - 122,152,308 |
| BLID | 122,115,340 - 122,116,215 |
| hsa-pri-miR-125b-1 | 122,099,757 - 122,099,844 |
| MIR99AHG | 15,928,296 - 16,645,467 |
| hsa-pri-miR-99a | 16,539,089 - 16,539,169 |
| hsa-pri-miR-125b-2 | 16,590,237 - 16,590,325 |
B
C

## Slide 3
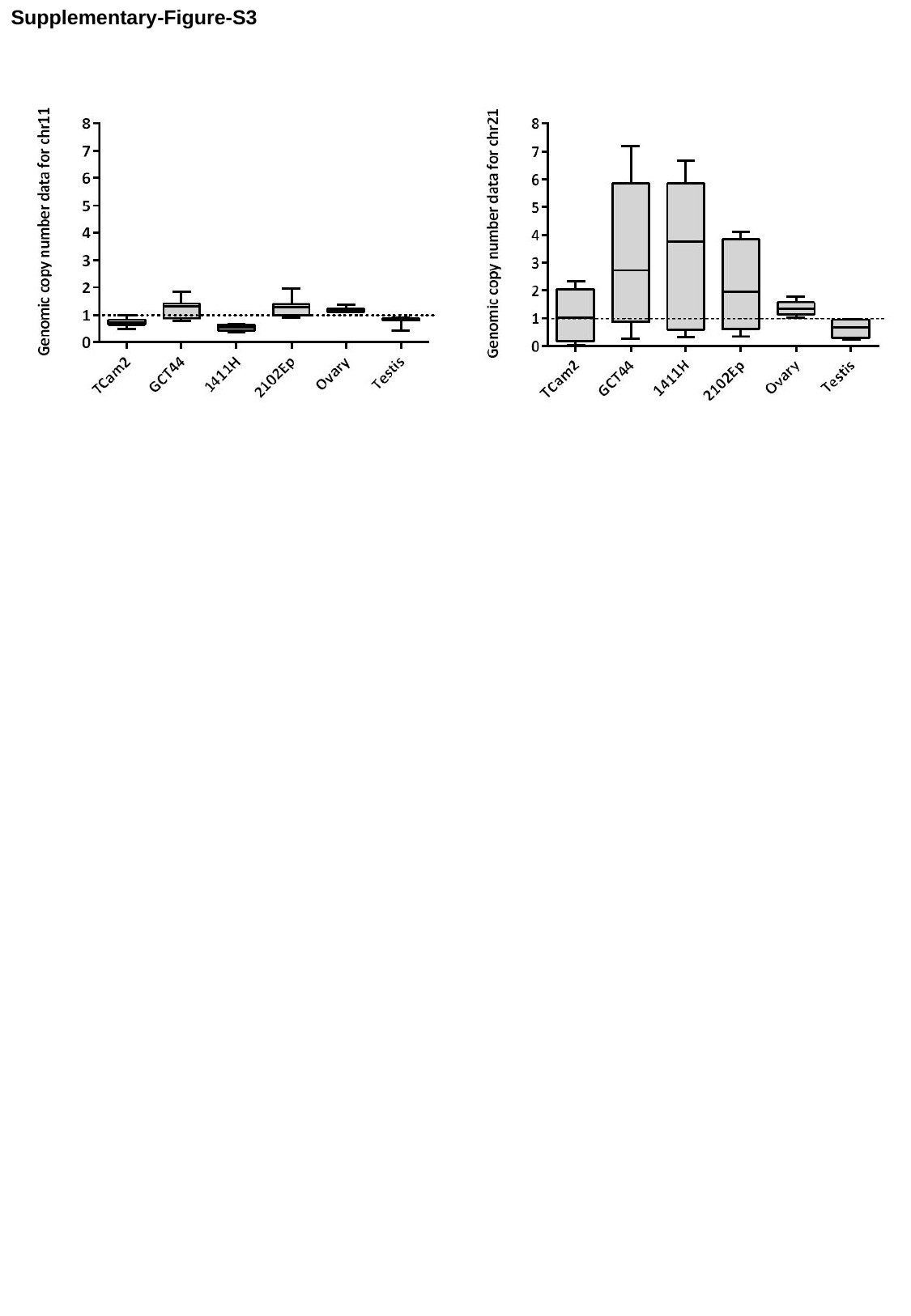

Supplementary-Figure-S3

## Slide 4
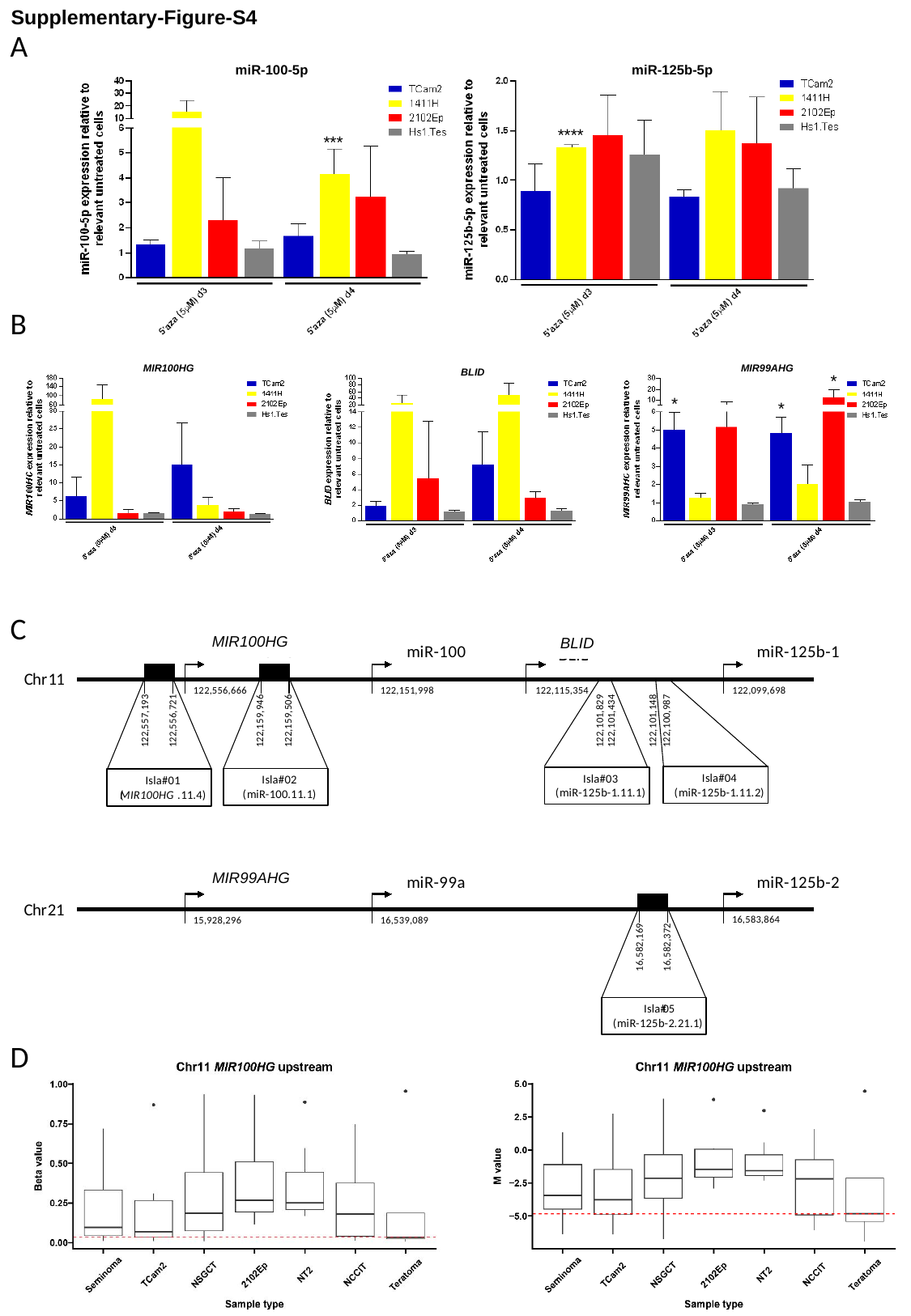

Supplementary-Figure-S4
A
miR-100-5p
miR-125b-5p
****
***
B
MIR100HG
MIR99AHG
BLID
*
*
*
C
MIR100HG
M
BLID
M
m
i
R
-
1
2
5
b
-
1
M
I
R
1
0
0
H
G
m
i
R
-
1
0
0
B
L
I
D
C
h
r
1
1
I
s
l
a
#
0
4
I
s
l
a
#
0
3
I
s
l
a
#
0
2
I
s
l
a
#
0
1
(
m
i
R
-
1
2
5
b
-
1
.
1
1
.
2
)
(
m
i
R
-
1
2
5
b
-
1
.
1
1
.
1
)
(
m
i
R
-
1
0
0
.
1
1
.
1
)
(
M
I
R
1
0
0
H
G
.
1
1
.
4
)
M
I
R
9
C
h
r
2
1
I
s
l
a
#
05
(
m
i
R
-
1
2
5
b
-
2
.
2
1
.
1
)
1
2
2
,
5
5
6
,
6
6
6
1
2
2
,
1
5
1
,
9
9
8
1
2
2
,
1
1
5
,
3
5
4
1
2
2
,
0
9
9
,
6
9
8
9
4
8
7
3
1
6
6
2
3
4
8
9
2
4
0
8
4
1
9
1
7
9
5
,
,
,
,
,
,
,
,
1
1
1
0
7
6
9
9
0
0
0
0
5
5
5
5
1
1
1
1
5
5
1
1
,
,
,
,
,
,
,
,
2
2
2
2
2
2
2
2
2
2
2
2
2
2
2
2
1
1
1
1
1
1
1
1
9
A
H
G
m
i
R
-
9
9
a
m
i
R
-
1
2
5
b
-
2
1
6
,
5
8
3
,
1
5
,
9
2
8
,
2
9
6
1
6
,
5
3
9
,
0
8
9
9
2
6
7
1
3
,
,
2
2
8
8
5
5
,
,
6
6
1
1
MIR99AHG
M
8
6
4
D

## Slide 5
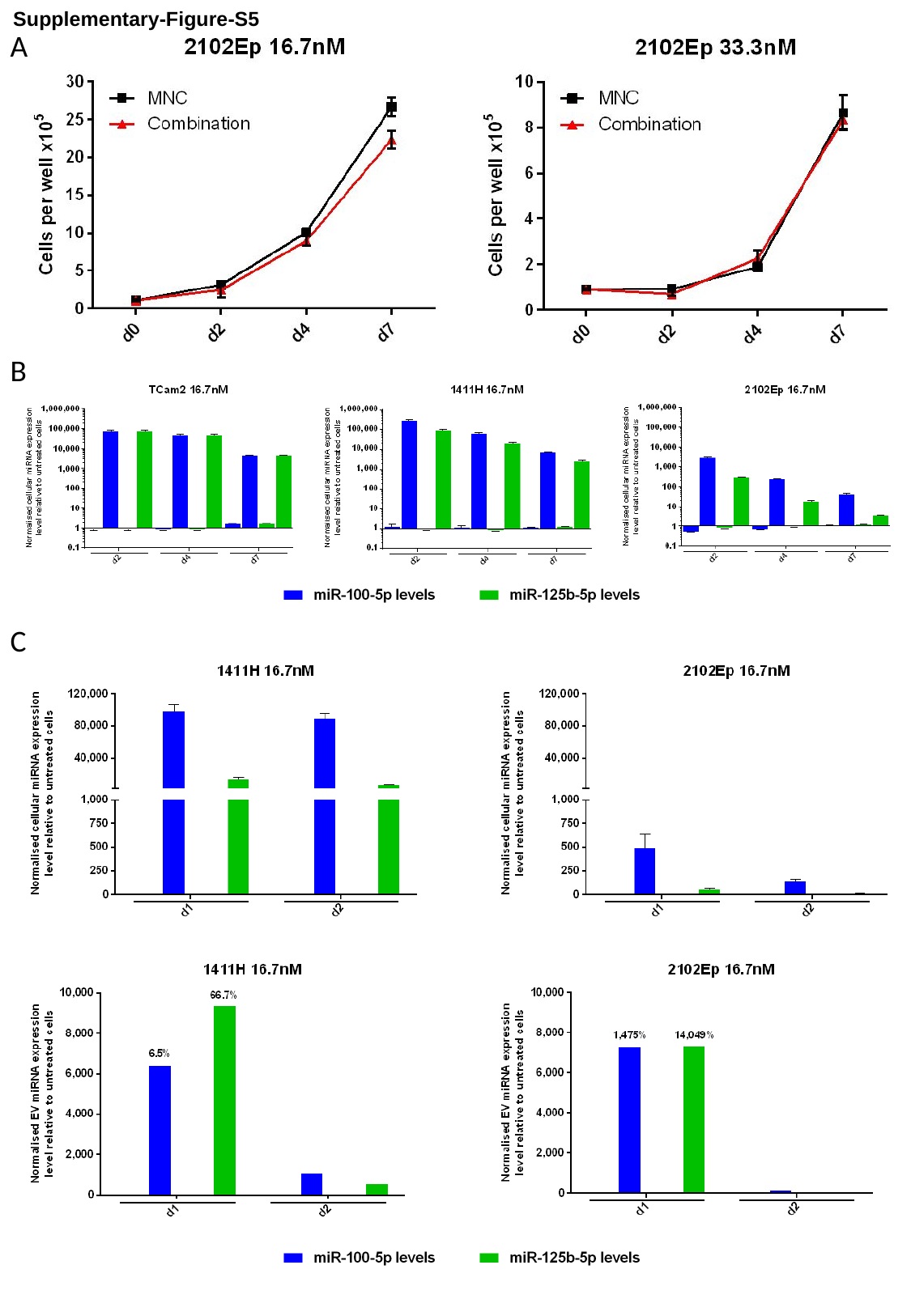

Supplementary-Figure-S5
A
B
C

## Slide 6
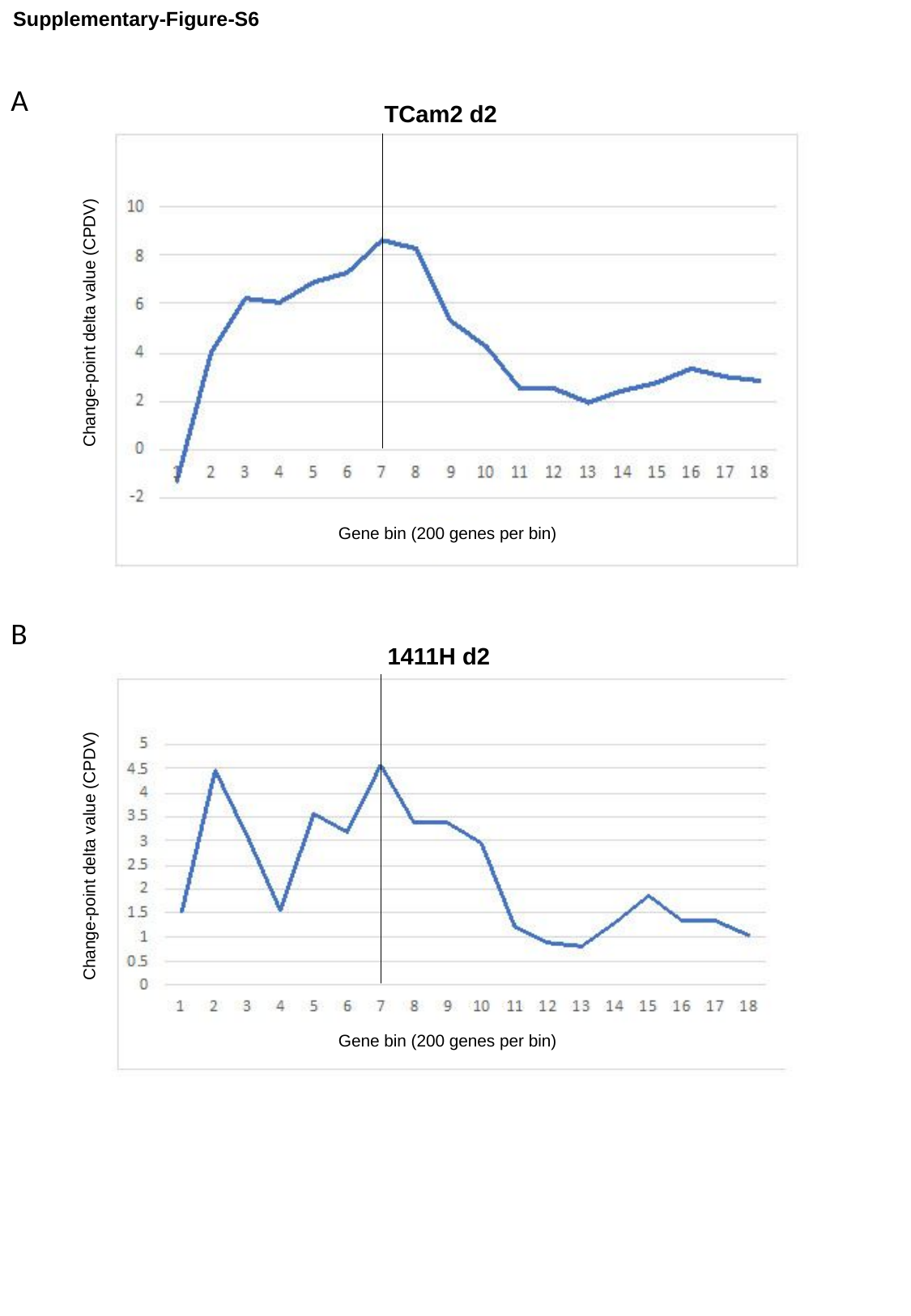

Supplementary-Figure-S6
A
TCam2 d2
Change-point delta value (CPDV)
Gene bin (200 genes per bin)
B
1411H d2
Change-point delta value (CPDV)
Gene bin (200 genes per bin)

## Slide 7
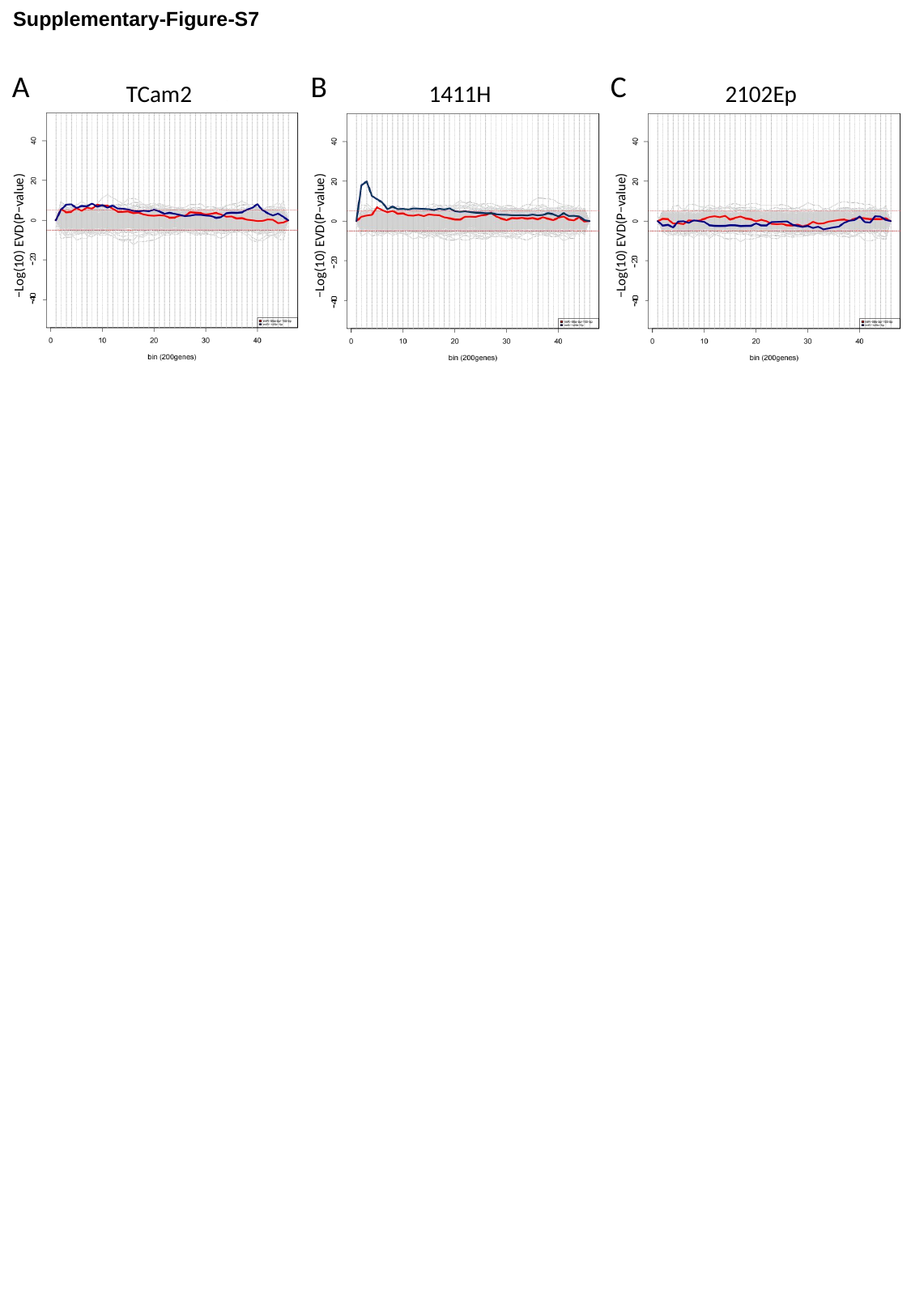

Supplementary-Figure-S7
A
B
1411H
-
-
−Log(10) EVD(P−value)
C
TCam2
−Log(10) EVD(P−value)
-
-
2102Ep
−Log(10) EVD(P−value)
-
-

## Slide 8
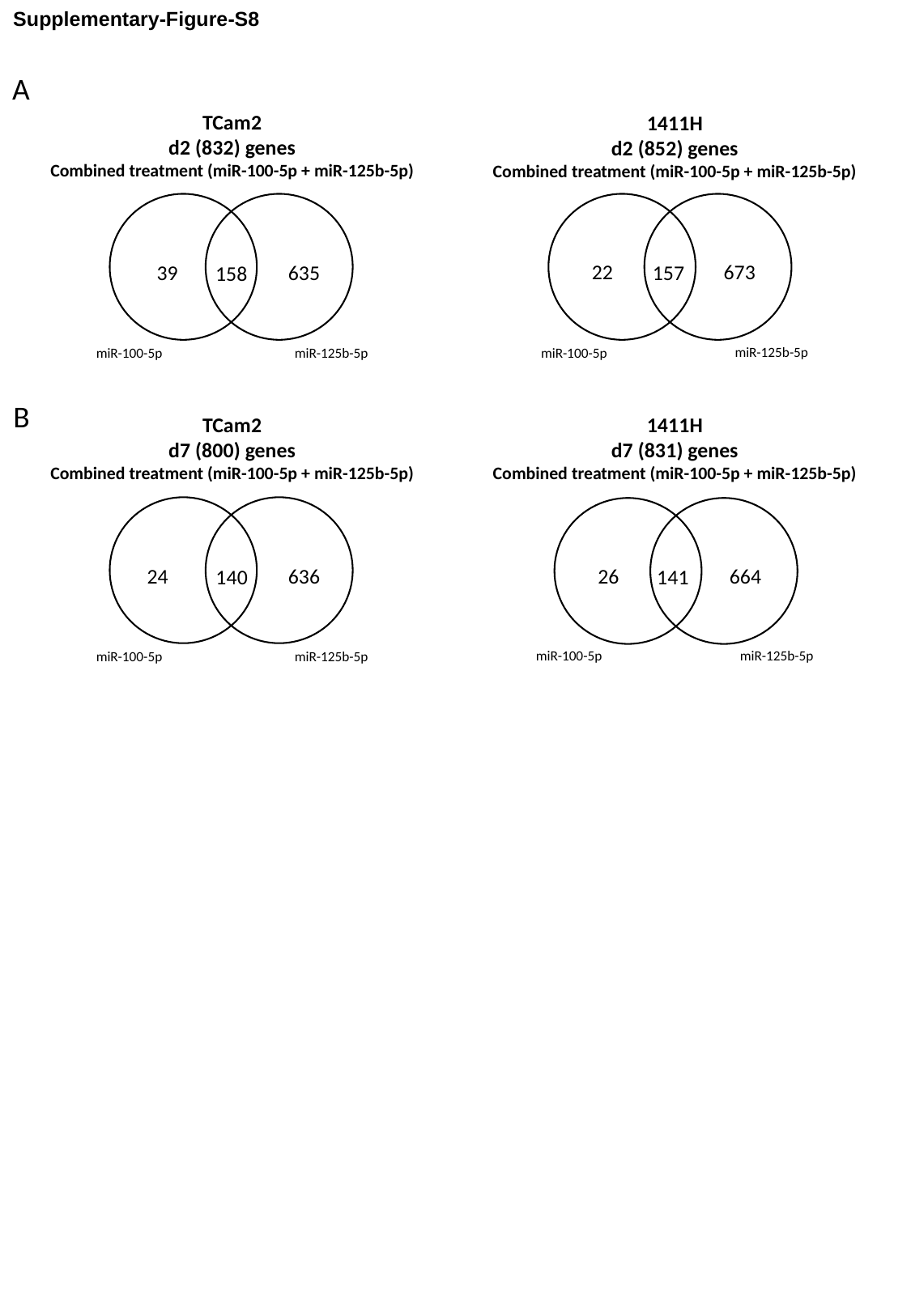

Supplementary-Figure-S8
A
TCam2
d2 (832) genes
Combined treatment (miR-100-5p + miR-125b-5p)
39
635
158
miR-100-5p
miR-125b-5p
1411H
d2 (852) genes
Combined treatment (miR-100-5p + miR-125b-5p)
22
673
157
miR-125b-5p
miR-100-5p
B
TCam2
d7 (800) genes
Combined treatment (miR-100-5p + miR-125b-5p)
24
636
140
miR-100-5p
miR-125b-5p
1411H
d7 (831) genes
Combined treatment (miR-100-5p + miR-125b-5p)
26
664
141
miR-100-5p
miR-125b-5p

## Slide 9
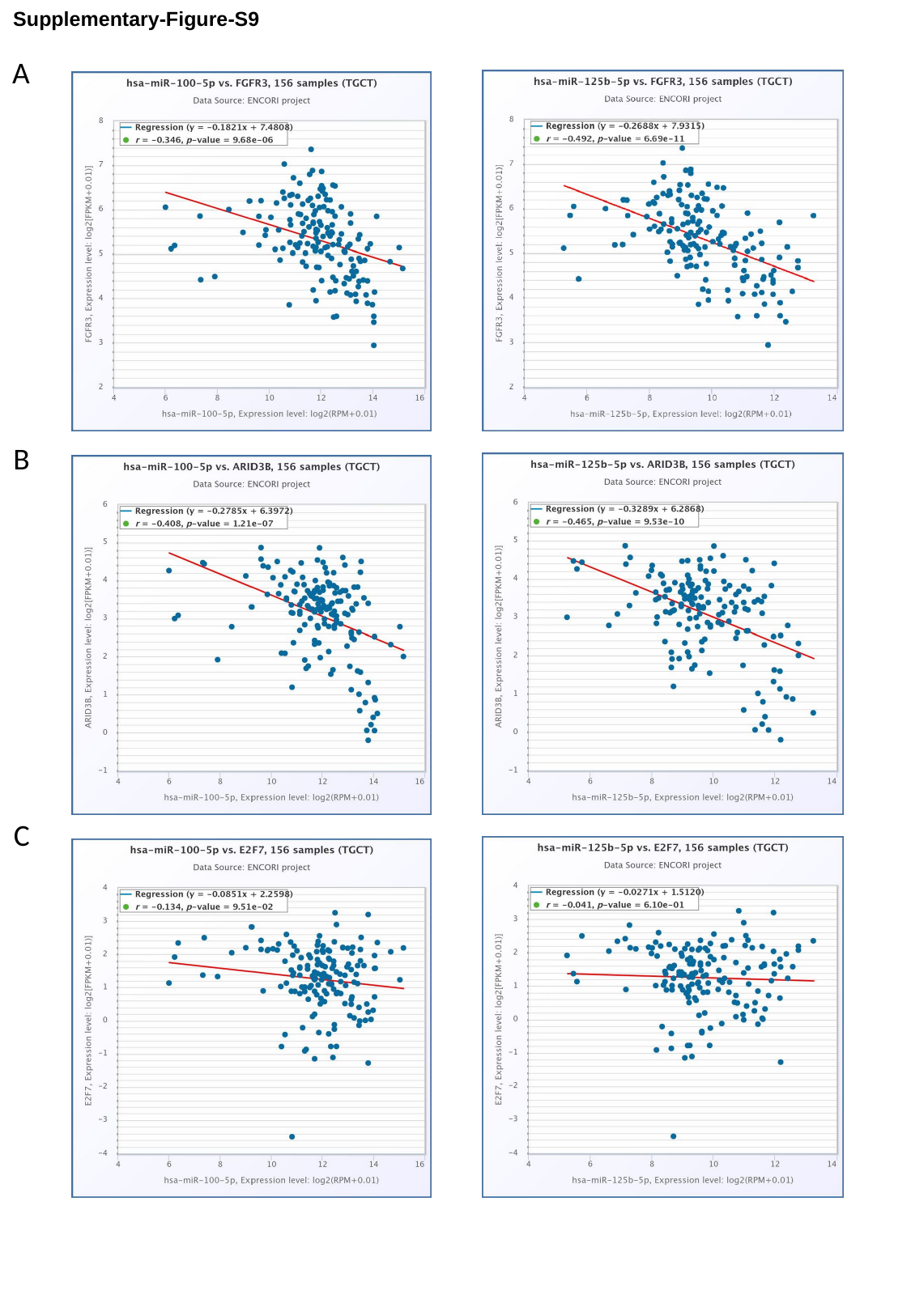

Supplementary-Figure-S9
A
B
C

## Slide 10
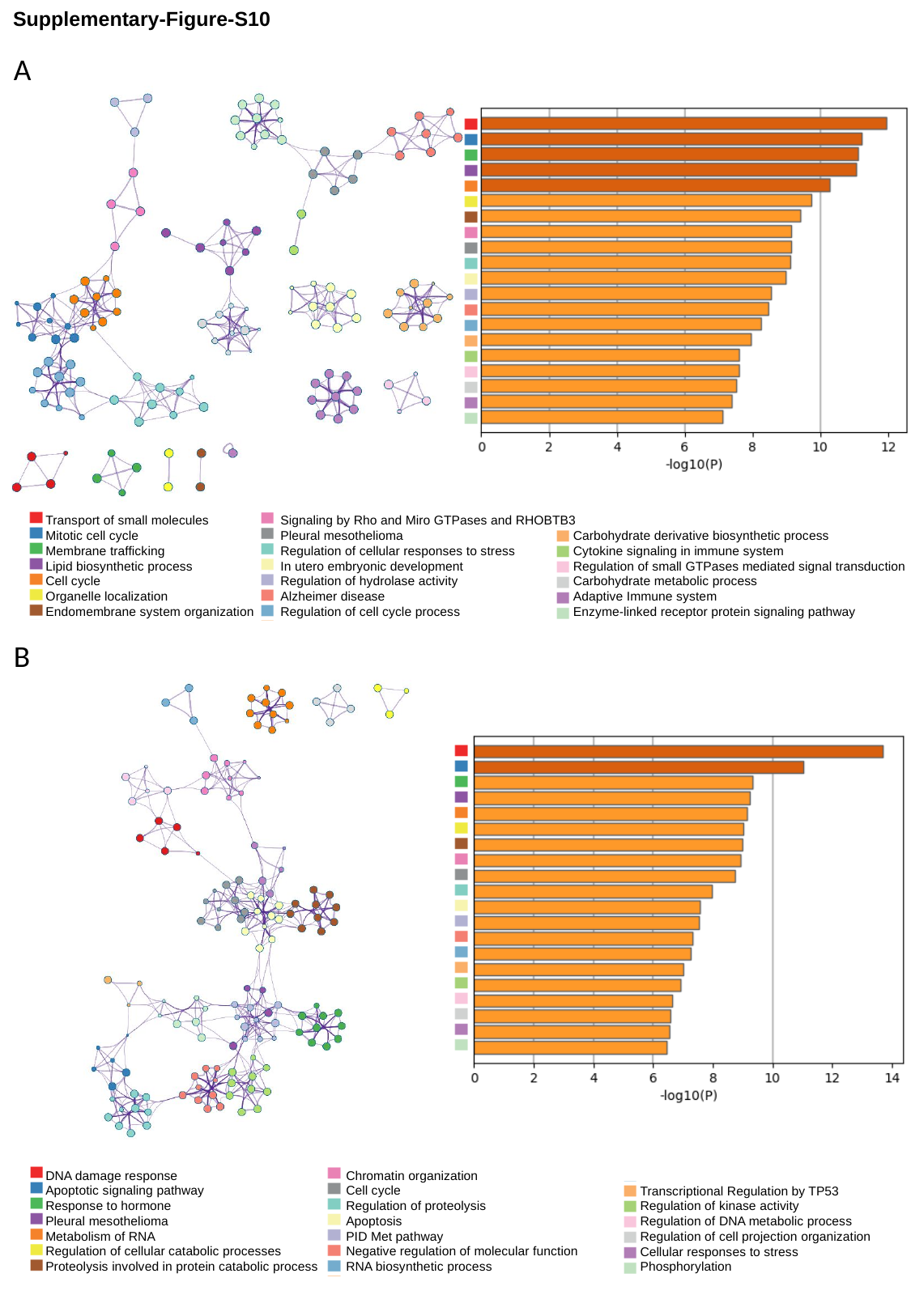

Supplementary-Figure-S10
A
Transport of small molecules
Mitotic cell cycle
Membrane trafficking
Lipid biosynthetic process
Cell cycle
Organelle localization
Endomembrane system organization
Signaling by Rho and Miro GTPases and RHOBTB3
Pleural mesothelioma
Regulation of cellular responses to stress
In utero embryonic development
Regulation of hydrolase activity
Alzheimer disease
Regulation of cell cycle process
Carbohydrate derivative biosynthetic process
Cytokine signaling in immune system
Regulation of small GTPases mediated signal transduction
Carbohydrate metabolic process
Adaptive Immune system
Enzyme-linked receptor protein signaling pathway
B
DNA damage response
Apoptotic signaling pathway
Response to hormone
Pleural mesothelioma
Metabolism of RNA
Regulation of cellular catabolic processes
Proteolysis involved in protein catabolic process
Chromatin organization
Cell cycle
Regulation of proteolysis
Apoptosis
PID Met pathway
Negative regulation of molecular function
RNA biosynthetic process
Transcriptional Regulation by TP53
Regulation of kinase activity
Regulation of DNA metabolic process
Regulation of cell projection organization
Cellular responses to stress
Phosphorylation

## Slide 11
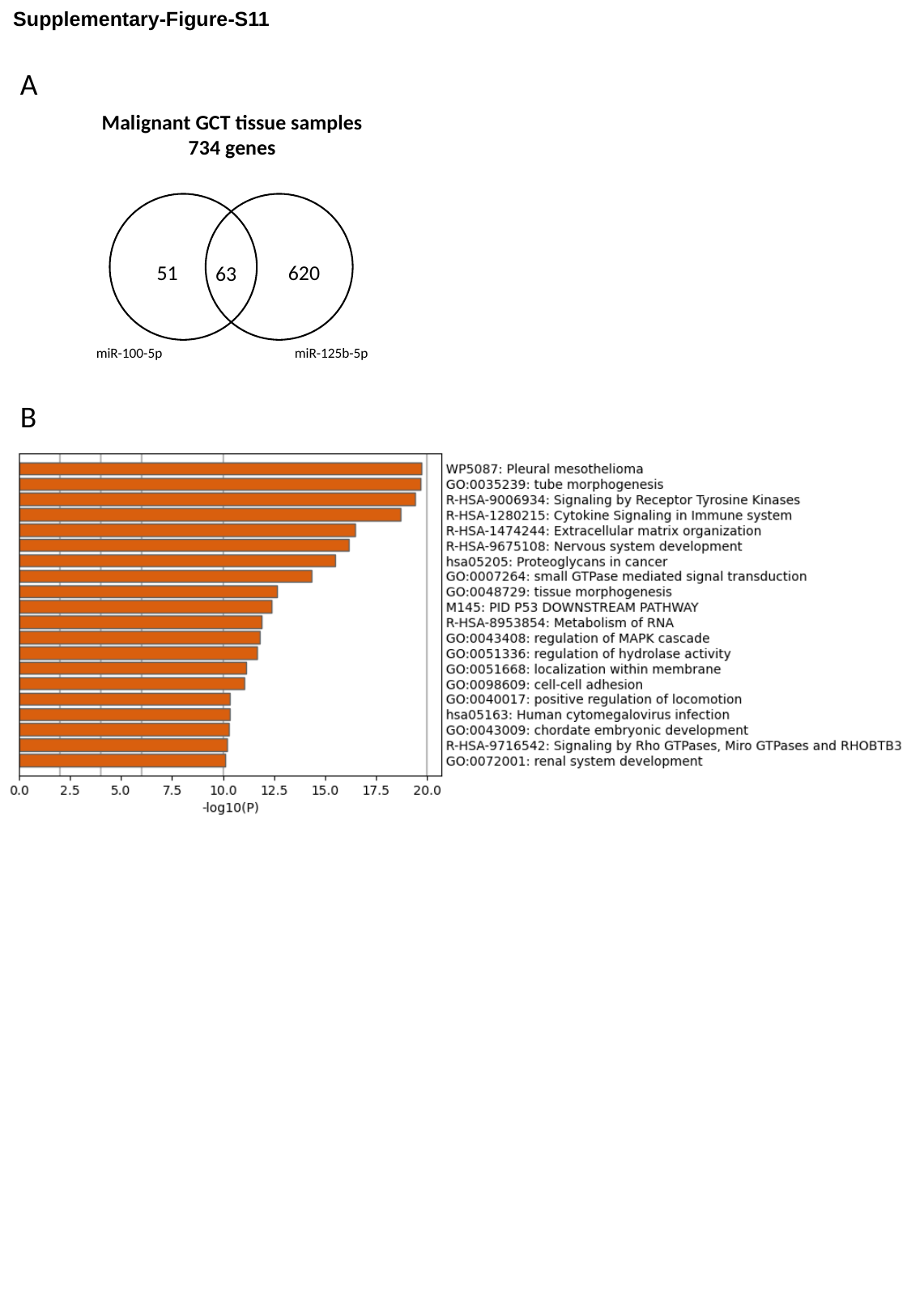

Supplementary-Figure-S11
A
Malignant GCT tissue samples
734 genes
51
620
63
miR-100-5p
miR-125b-5p
B
