# Supplementary material for: Replenishing co‐downregulated miR‐100‐5p and miR‐125b‐5p in malignant germ cell tumors causes growth inhibition through cell cycle disruption
Source: Mol Oncol. 2024 Nov 10;19(4):1203–28. doi: 10.1002/1878-0261.13757 (PMC11977657; doi:10.1002/1878-0261.13757)
Supplement: Supplementary file 2 — Table S1. Clinico‐pathological data for the germ cell tumor (GCT) pediatric clinical samples and GCT cell lines used in the study. Table S2. Primers used for genomic DNA (gDNA) copy number assessment across the regions of interest on chromosomes 11 and 21 in malignant germ cell tumor (GCT) cell lines and normal testis. Table S3. Initial polymerase chain reaction (PCR) screening across the upstream regions of interest of the genes on (A) chromosome 11 (11q24.1) and (B) chromosome 21 (21q21.1). Table S4. List of primers used for formal methylation assessment of the regions of interest on (A) chromosome 11 (11q24.1) and (B) chromosome 21 (21q21.1). Table S5. Illumina messenger RNA (mRNA) microarray information. Table S6. Elements comprising the seed complementary regions (SCRs) for the microRNA (miRNA) seed regions of interest for derivation of the Sylamer single summed significance score (SSSS). Table S7. Downregulation of miR‐99a‐5p, miR‐100‐5p, and miR‐125b‐5p in malignant germ cell tumors (GCTs), GCT subtypes, and cell lines relative to non‐malignant (gonadal and teratoma) control samples; derived from global microRNA (miRNA) microarray expression data. Table S8. Enrichment for seed complementary regions (SCRs) corresponding to the seed regions of miR‐99a‐5p/miR‐100‐5p and miR‐125b‐5p in 3′ untranslated regions (3′UTRs) of messenger RNA (mRNA) data. Table S9. The top‐20 deregulated Metascape pathways in TCam2 cells at day 2 (d2) after combination miR‐100‐5p/miR‐125b‐5p replenishment. Table S10. The top‐20 deregulated Metascape pathways in 1411H cells at day 2 (d2) after combination miR‐100‐5p/miR‐125b‐5p replenishment. Table S11. The top‐20 deregulated Metascape pathways in TCam2 cells at day 7 (d7) after combination miR‐100‐5p/miR‐125b‐5p replenishment. Table S12. The top‐20 deregulated Metascape pathways in 1411H cells at day 7 (d7) after combination miR‐100‐5p/miR‐125b‐5p replenishment. [file MOL2-19-1203-s003.docx]

**Supplementary Information:**

- Supplementary Tables S1-S12 with Legends; Legends to Supplementary Tables S13-S16 (see separate excel files for these Tables)
- Legends to Supplementary Figures S1-S11
- Supplementary References

**Supplementary Tables**

**Supplementary-Table-S1**

| **Sample** | **Sample ID** | **Tumor**  **Subtype** | **Tumor site** | **Age (years)** | **Sex** | **TNM stage** | **Follow-up** | **Global miRNA array** | **miRNA qRT-PCR validation** | **Global mRNA array** |
| --- | --- | --- | --- | --- | --- | --- | --- | --- | --- | --- |
| 1 | 16/239  (YST-1) | YST (Mixed, with MT) | CNS | 12 | M | 1 | NED | ✓ |  | ✓ |
| 2 | 21/53  (YST-2) | YST  (Mixed, with MT) | Sacrococcygeal | 3 | F | 4 | NED | ✓ |  | ✓ |
| 3 | 14/41  (YST-3) | YST  (Pure) | Testis | 2 | M | 1 | NED | ✓ | ✓ | ✓ |
| 4 | 15/5  (YST-4) | YST  (Pure) | Ovary | 13 | F | 3 | NED | ✓ | ✓ | ✓ |
| 5 | 6/11  (YST-5) | YST  (Pure) | Ovary | 12 | F | 1 | NED | ✓ |  | ✓ |
| 6 | 5/49  (YST-6) | YST  (Pure) | Testis | 4 | M | 4 | NED | ✓ |  | ✓ |
| 7 | 6/117  (YST-7) | YST  (Pure) | Testis | 0 | M | 1 | NED | ✓ |  | ✓ |
| 8 | 22/24  (YST-8) | YST  (Mixed, with MT) | Sacrococcygeal | 2 | F | 4 | NED | ✓ |  | ✓ |
| 9 | 6/251  (YST-9) | YST  (Pure) | Ovary | 4 | F | 4 | NED | ✓ | ✓ |  |
| 10 | 12/65  (YST-10) | YST  (Pure) | Ovary | 0 | F | 2 | REL | ✓ | ✓ | ✓ |
| 11 | 5/91  (YST-11) | YST  (Pure) | Ovary | 14 | F | 1 | REL | ✓ | ✓ | ✓ |
| 12 | 18/194  (YST-12) | YST  (Pure) | Vagina | 1 | F | 1 | NED | ✓ | ✓ |  |
| 13 | 12/3 | YST | Ovary | 14 | F | 4 | NED |  | ✓ |  |
| 14 | 21/4 | YST | Ovary | 12 | F | 2 | N/A |  | ✓ |  |
| 15 | 12/245 | YST  (Mixed, with MT) | Sacrococcygeal | 1 | M | 4 | NED |  | ✓ |  |
| 16 | 4/144  (Sem-13) | Seminoma  (Pure) | Ovary | 12 | F | 1 | DOD | ✓ |  | ✓ |
| 17 | 16/241  (Sem-14) | Seminoma  (Pure) | CNS (Pineal) | 10 | F | 1 | DOD | ✓ |  | ✓ |
| 18 | 16/240  (Sem-15) | Seminoma  (Pure) | CNS (Pineal) | 16 | F | 1 | NED | ✓ |  | ✓ |
| 19 | 4/546  (Sem-16) | Seminoma  (Pure) | CNS (Pineal) | 12 | M | 1 | DOD | ✓ |  |  |
| 20 | TB254  (Sem-17) | Seminoma  (Pure) | Testis | 25 | M | 1 | NED | ✓ | ✓ |  |
| 21 | TB608  (Sem-18) | Seminoma  (Pure) | Testis | 35 | M | 1 | NED | ✓ | ✓ |  |
| 22 | 5/277  (Sem-19) | Seminoma  (Pure) | Ovary | 8 | F | N/A | NED | ✓ | ✓ |  |
| 23 | 16/268  (Sem-20) | Seminoma  (Pure) | Ovary | 9 | F | 3 | NED | ✓ | ✓ |  |
| 24 | 6/212  (Sem-21) | Seminoma  (Pure) | Lymph node metastasis of ovarian primary | 11 | F | 4 | REL | ✓ | ✓ |  |
| 25 | 15/111  (Sem-22) | Seminoma  (Pure) | Ovary | 14 | F | 3 | NED | ✓ | ✓ |  |
| 26 | 10/128  (Sem-23) | Seminoma  (Pure) | Ovary | 13 | F | 3 | NED | ✓ |  | ✓ |
| 27 | 5/154  (Sem-24) | Seminoma  (Pure) | Ovary | 12 | F | 1 | NED | ✓ | ✓ | ✓ |
| 28 | 011214  (Sem-25) | Seminoma  (Pure) | Ovary | 13 | F | 1 | REL | ✓ | ✓ | ✓ |
| 29 | 4/71  (EC-26) | EC  (Pure) | Lung metastasis of testicular primary | 15 | M | 4 | REL | ✓ | ✓ | ✓ |
| 30 | 21/84  (EC-27) | EC  (Pure) | Testis | 15 | M | N/A | N/A | ✓ |  |  |
| 31 | 6BT  (EC-28) | EC  (Pure) | Testis | 13 | M | 4 | NED | ✓ | ✓ |  |
| 32 | 1189743 DU  (IT-29) | Teratoma  (Pure) | Ovary | 0 | F | Immature | NED | ✓ |  |  |
| 33 | 21/50  (IT-30) | Teratoma  (Pure) | Ovary | 15 | F | Immature | NED | ✓ | ✓ |  |
| 34 | 5/166  (IT-31) | Teratoma  (Pure) | Abdominal | 0 | M | Immature | NED | ✓ | ✓ |  |
| 35 | 14/202 | Teratoma | Ovary | 11 | F | Immature | NED |  | ✓ |  |
| 36 | 3/21  (MT-32) | Teratoma  (Pure) | Sacrococcygeal | 0 | F | Mature | NED | ✓ |  |  |
| 37 | 4/428  (MT-33) | Teratoma  (Pure) | Ovary | 3 | F | Mature | NED | ✓ | ✓ |  |
| 38 | 12/95  (MT-34) | Teratoma  (Mixed, with YST) | Ovary | 12 | F | Mature | NED | ✓ |  |  |
| 39 | 16/312 | Teratoma | Mediastinum | 2 | F | Mature | NED |  | ✓ |  |
| 40 | Tera2  (CL-35) | Teratoma | Testis-derived cell line | N/A | N/A | N/A | N/A | ✓ | ✓ |  |
| 41 | GCT44  (CL-36) | YST | Testis-derived cell line | N/A | N/A | N/A | N/A | ✓ | ✓ |  |
| 42 | PA-1  (CL-37) | Teratoma | Ovary-derived cell line | N/A | N/A | N/A | N/A | ✓ | ✓ |  |
| 43 | 1411H  (CL-38) | YST | Abdominal metastasis-derived cell line | N/A | N/A | N/A | N/A | ✓ |  |  |
| 44 | TCam2  (CL-39) | Seminoma | Testis-derived cell line | N/A | N/A | N/A | N/A | ✓ | ✓ |  |
| 45 | 2102Ep  (CL-40) | EC | Testis-derived cell line | N/A | N/A | N/A | N/A | ✓ | ✓ |  |
| 46 | NCCIT | EC | Testis-derived cell line | N/A | N/A | N/A | N/A |  | ✓ |  |
| 47 | Prepubescent testis T1  (N-41) | Normal | Testis | N/A | N/A | N/A | N/A | ✓ |  | ✓ |
| 48 | Prepubescent ovary O1  (N-42) | Normal | Ovary | N/A | N/A | N/A | N/A | ✓ |  |  |
| 49 | Postpubescent testis T2  (N-43) | Normal | Testis | N/A | N/A | N/A | N/A | ✓ |  | ✓ |
| 50 | Postpubescent ovary O2  (N-44) | Normal | Ovary | N/A | N/A | N/A | N/A | ✓ |  | ✓ |
| 51 | 0811  (D-45) | Normal | Fetal  yolk sac | N/A | N/A | N/A | N/A | ✓ |  |  |
| 52 | N859  (D-46) | Normal | Fetal  yolk sac | N/A | N/A | N/A | N/A | ✓ |  |  |
| 53 | N868  (D-47) | Normal | Fetal ovary | N/A | N/A | N/A | N/A | ✓ |  |  |
| 54 | N858  (D-48) | Normal | Fetal ovary | N/A | N/A | N/A | N/A | ✓ |  |  |
| 55 | Gonadal reference (Ovary) | Normal | Ovary - Ambion (AM6974) | N/A | N/A | N/A | N/A |  | ✓ |  |
| 56 | Gonadal reference (Testis) | Normal | Testis - Ambion (AM7972) | N/A | N/A | N/A | N/A |  | ✓ |  |

**Supplementary-Table-S1. Clinico-pathological data for the germ cell tumor (GCT) pediatric clinical samples and GCT cell lines used in the study.** The table reports available clinico-pathological data from the biobanks from which the samples were derived. It should be noted that the four yolk sac tumors (YSTs) which were part of mixed GCTs comprised a substantial YST component that was sampled alone, without the need for microdissection. Additional information is provided to clarify which samples underwent global microRNA (miRNA) array profiling (total n=48), additional miRNA validation (total n=32) and global messenger RNA (mRNA) array (total n=20). Key: YST = yolk sac tumor; EC= embryonal carcinoma; CNS = central nervous system; NED = no evidence of disease; DOD = died of disease; REL = relapsed; TNM = tumor-node-metastasis; N/A = not available.

**Supplementary-Table-S2**

| CHROMOSOME 11 | | |
| --- | --- | --- |
| **Name** | **Forward primer (5’ to 3’)** | **Reverse Primer (5’ to 3’)** |
| 50Kb upstream miR-100 | GTGTATGGGGAAGGCAATGT | GCACGAAGGAAGGATGGTAA |
| 10Kb upstream miR-100 | CTAGCCATGTTGCTTGTTTCTTT | AGACACCTTTCATTCTACCACCA |
| 5Kb upstream miR-100 | GATAAAACGTGGCACGACAAT | TTTGCCAAAAGATCCAATCAC |
| miR-100 | CCCGTAGATCCGAACTTGTG | TATAAGCAAAGCCCCAGGTC |
| *let-7a-2* | ATGCTCCCAGGTTGAGGTAGTA | GTTTAGTGCAAGACCCAAGGAA |
| 10Kb upstream miR-125b-1 | CGGCTAGATTGAATGAAAGACC | CATAGTTGGGAACTGAGGCAAT |
| miR-125b-1 | AGAAAACATTGTTGCGCTCCT | AGAGCCTAACCCGTGGATTTA |
| **CHROMOSOME 21** | | |
| **Name** | **Forward primer (5’ to 3’)** | **Reverse Primer (5’ to 3’)** |
| 50Kb upstream miR-99a | TGAAAACATTGTGTCAGCTTCA | TTAATCCGCAGAGGCAATTC |
| 10Kb upstream miR-99a | AAATTGTGAAACGTTCCCTCTTC | GCATCTGGCCATTATTGAAAGT |
| 5Kb upstream miR-99a | GATAAAACGTGGCACGACAAT | TTTGCCAAAAGATCCAATCAC |
| miR-99a | CCCGTAGATCCGAACTTGTG | TATAAGCAAAGCCCCAGGTC |
| *let-7c* | ATGCTCCCAGGTTGAGGTAGTA | GTTTAGTGCAAGACCCAAGGAA |
| 10Kb upstream miR-125b-2 | CGGCTAGATTGAATGAAAGACC | CATAGTTGGGAACTGAGGCAAT |
| miR-125b-2 | AGAAAACATTGTTGCGCTCCT | AGAGCCTAACCCGTGGATTTA |
| **HOUSEKEEPING GENES** | | |
| **Name** | **Forward primer (5’ to 3’)** | **Reverse Primer (5’ to 3’)** |
| *GAPDH* | TTGTCAAGCTCATTTCCTGGT | GAATGAGGAACCTCCGGTACA |
| *B2M* | CGGCTCTGCTTCCCTTAGACT | TCACAGCCAAGCATTCTACAAAC |
| 18A | GAGGCACCTCTCGTTTCAAG | TTCTCCCTAAGCCTTCACGA |
| 18B | GCAGTAGGGCAAGGGTGTAG | TGGAGGTTTGGATTGAGGTC |

**Supplementary-Table-S2. Primers used for genomic DNA (gDNA) copy number assessment across the regions of interest on chromosomes 11 and 21 in malignant germ cell tumor (GCT) cell lines and normal testis.**

**Supplementary-Table-S3**

| 1. **CHROMOSOME 11 (11q24.1) – SCREENING ACROSS THE REGION OF INTEREST (ROI)** | | |
| --- | --- | --- |
| Name | Forward primer (5’ to 3’) | Reverse Primer (5’ to 3’) |
| MIR100HG up to 10Kb upstream #1 | TTTTTTTATTATATGTTAATTTTTTTAGTT | AAACACATTCTAATATTCTCTACCATTATA |
| MIR100HG up to 10Kb upstream #2 | GGTTAGGAGTGGTGGTTGGA | TCCTACCTCAACCTTACTAAACAAC |
| MIR100HG up to 10Kb upstream #3 | GTTGAGGTAGGAGAATTGTTTGAAT | CCACTAAACCCCACTAACTTTAATAAA |
| MIR100HG up to 5Kb upstream #4 | AGAGGGTTGGTTGGTGTATTT | TCACTCTCTACCCCTCCTCA |
| miR-100 up to 10Kb upstream #5 | AGGGGTATGGAGAAATGAAGGA | ACTCAACTCCCATTTCACTCAA |
| miR-100 up to 5Kb upstream #6 | TTATTTGAAATGAAAAATTTTTTTT | TTATAATAAAACAAAATAAACCCAAC |
| miR-125b-1 up to 5Kb upstream #1 | GGGGTAGTGTGGTTTAGGAAA | CCCCACCTTCAAAACAACTTT |
| miR-125b-1 up to5Kb upstream #2 | TTTATTTTTAGTTTGATGAGGAAAG | CACCAAACTATCATTTAATAAACAC |
| miR-125b-1 up to 5Kb upstream #3 | GATTTTAGTTAAGTAGTTGATGTAGA | AACCTCCTATAACACCATCC |
| miR-125b-1 up to 5Kb upstream #4 | GGATGGTGTTATAGGAGGTT | CTTTCCTTCACAAAATTAAAATTC |
| BLID up to 5Kb upstream | GGAGTGTAGTGGTGTGATTTTA | AACAACATACAAAAAATACATCCTA |

| **B) CHROMOSOME 21 (21q21.1) – SCREENING ACROSS THE REGION OF INTEREST (ROI)** | | |
| --- | --- | --- |
| Name | Forward primer (5’ to 3’) | Reverse Primer (5’ to 3’) |
| MIR99AHG up to 10Kb upstream | TGGGTATTAGTAGTGGTGGTTGTAG | CTACCTCCTCAAATAAATCCCTAAC |
| miR-99a up to 5Kb upstream | TTAAAATTGTTATTGTGAAAATAATA | TCTAAAAAAAACATCTAAATAAATC |
| miR-99a up to 10Kb upstream #1 | GGTTGAAATTTGGATATTTTAGGTATTAG | AAACCACAATAAAAACAACAACAAAC |
| miR-99a up to 10Kb upstream #2 | GTTAATGAATGTGTTATTTGTTTT | ATTACCCCTTTTCCATATATAAAC |
| miR-125b-2 up to 5Kb upstream | TTGTAGTTTAGTAGGTTTGGGAGAG | TAACTAACTAACTTTTACCCTCCAC |

**Supplementary-Table-S3**. **Initial polymerase chain reaction (PCR) screening across the upstream regions of interest of the genes on A) chromosome 11 (11q24.1) and B) chromosome 21 (21q21.1).**

**Supplementary-Table-S4**

| **A) CHROMOSOME 11 (11q24.1) – METHYLATION ASSESSMENT ACROSS THE REGION OF INTEREST (ROI)** | | | |
| --- | --- | --- | --- |
|  | **PCR template** | **Pyrosequencing** | |
| **Area** | **Forward (F) and Reverse (R) primers (5’ to 3’)** | **Biotinylated primer** | **Assay primer** |
| Up to 5Kb upstream of MIR100HG  #4  122159471 - 122159954 | AGAGGGTTGGTTGGTGTATTT (F)  TCACTCTCTACCCCTCCTCA (R) | TCACTCTCTACCCCTCCTCA | 1. TAAATTTTTAGTTAGATATAGAAT  2. ATAGAAAAGTTTTTTAAGTTTTTAT  3. TAATGGTATTTGTAGTGGGA  4. GGTTTTTTAGTAGTTTAAAGG |
|  |  | AGAGGGTTGGTTGGTGTATTT | 5. CAACTAACTCRATTTCTAAAT  6. AACCCCTCTCTAAAACTAACT  7. CTCTCTACCCCTCCTCAAC |
| Up to 10Kb upstream of miR-100 #5  122556672 - 122557137 | AGGGGTATGGAGAAATGAAGGA (F)  ACTCAACTCCCATTTCACTCAA (R) | ACTCAACTCCCATTTCACTCAA | 1. GTTAAGTTTGAAAGATGATGA  2. GATTAATGTAGAAGGTTGG  3. GATTAGAATATAAGATGGA |
| Up to 5Kb upstream of miR-125b-1 #1  122101418 - 122101833 | GGGGTAGTGTGGTTTAGGAAA (F)  CCCCACCTTCAAAACAACTTT (R) | CCCCACCTTCAAAACAACTTT | 1. GTGTGGTTTAGGAAAATTT  2. GATTAAATGTGTTTTTTAAAG  3. AATGGTAAAAGAGAAAAGG  4. AAGGGAGAGAAAAAGGAGAG |
| Up to 5Kb upstream of miR-125b-1 #2  122100814 - 122101023 | TTTATTTTTAGTTTGATGAGGAAAG (F)  CACCAAACTATCATTTAATAAACAC (R) | CACCAAACTATCATTTAATAAACAC | 1. TTTTTAGTTTGATGAGGAAA  2. GGTTAAAGGTTTTAAAGAATT |
| **B) CHROMOSOME 21 (21q21.1) – METHYLATION ASSESSMENT ACROSS THE REGION OF INTEREST (ROI)** | | | |
|  | **PCR template** | **Pyrosequencing** | |
| **Area** | **Forward (F) and Reverse (R) primers (5’ to 3’)** | **Biotinylated primer** | **Assay primer** |
| Up to 5Kb upstream of miR-125b-2 #16  16588482 - 16588742 | TTGTAGTTTAGTAGGTTTGGGAGAG (F)  TAACTAACTAACTTTTACCCTCCAC (R) | TAACTAACTAACTTTTACCCTCCAC | 1. GAGTTAATGTTAATGATGAG  2. GTTTTGAGTTAGTATTAGAT |

**Supplementary-Table-S4. List of primers used for formal methylation assessment of the regions of interest on A) chromosome 11 (11q24.1) and B) chromosome 21 (21q21.1).** Ensembl positions of the relevant regions of interest are listed in the ‘Area’ column. Note that an ‘R’ in the primer sequence means that the primers at that base were a mixture of cytosine (C) and thymine (T) bases, to allow for unbiased amplification of both methylated and unmethylated regions. (F) = forward primer; (R) = reverse primer.

**Supplementary-Table-S5**

| **Illumina mRNA microarray probe and gene information** | | | | |
| --- | --- | --- | --- | --- |
| *Probe/gene information* | *TCam2 d2* | *1411H d2* | *TCam2 d7* | *1411H d7* |
| Total number of differentially expressed Illumina probes | 18,960 | 18,930 | 19,662 | 19,749 |
| Total number of unique genes with known 3'UTR information | 8,706 | 8,702 | 9,004 | 8,953 |

**Supplementary-Table-S5. Illumina messenger RNA (mRNA) microarray information.** Summary of the number of differentially expressed genes identified within the Illumina microarray and the total number of unique genes with known 3’ untranslated region (3’UTR) information at different conditions/timepoints [day 2 (d2) and day 7 (d7)] for the combination replenishment of miR-100-5p and miR-125b-5p experiments.

**Supplementary-Table-S6**

| 1. **The six SCR elements of miR-99a-5p/miR-100-5p** | | |
| --- | --- | --- |
| *1-6nt hexamer:* TACGGG | *2-7nt hexamer:* ACGGGT | *3-8nt hexamer:* CGGGTA |
| *1-7nt heptamer:* TACGGGT | *2-8nt heptamer:* ACGGGTA |  |
| *1-8nt octamer:* TACGGGTA |  |  |
| 1. **The six SCR elements of miR-125b-5p** | | |
| *1-6nt hexamer:* CTCAGG | *2-7nt hexamer:* TCAGGG | *3-8nt hexamer:* CAGGGA |
| *1-7nt heptamer:* CTCAGGG | *2-8nt heptamer:* TCAGGGA |  |
| *1-8nt octamer:* CTCAGGGA |  |  |

**Supplementary-Table-S6. Elements comprising the seed complementary regions (SCRs) for the microRNA (miRNA) seed regions of interest for derivation of the *Sylamer* single summed significance score (SSSS).** The SSSS was an integration of *Sylamer* significance scores for different elements that comprised the SCR and served as an overall evaluation of the enrichment or depletion of nucleotide (nt) sequences. For this work, the scores were calculated by combining the *Sylamer* results for six SCR elements, all complementary to the 1-8nt miRNA seed region, namely three hexamers (1-6nt, 2-7nt, and 3-8nt), two heptamers (1-7nt and 2-8nt) and one octamer (1-8nt) for A) miR-99a-5p/miR-100-5p and B) miR-125b-5p. Note that due to overrepresentation of conserved adenosines flanking SCRs in messenger RNAs (mRNAs), the complementarity criterion was discarded for SCR position 8 (seed position 1), where the nucleotide was always set to be adenosine, irrespective of the actual nucleotide at that position, as described (1). Accordingly, the miR-99a-5p/miR-100-5p 1-8nt SCR was TACGGGTA, rather than TACGGGTT, also reflected in the 2-8nt heptamer and 3-8nt hexamer.

**Supplementary-Table-S7**

| **miRNA** | **All malignant GCT tissues** | | |
| --- | --- | --- | --- |
|  | Downregulated rank (n=126) | Log_2_ FC | Adjusted *p*-value |
| miR-99a-5p | 4 | -3.47 | 9.25 E-08 |
| miR-100-5p | 5 | -3.39 | 1.23 E-07 |
| miR-125b-5p | 15 | -3.45 | 9.59 E-07 |
| (miR-99b-5p) | (10) | (-1.80) | (3.24 E-07) |
| (miR-125a-5p) | (20) | (-1.92) | (1.30 E-06) |
|  | | | |
| **miRNA** | **Germinoma / Seminoma** | | |
|  | Downregulated rank (n=93) | Log_2_ FC | Adjusted *p*-value |
| miR-99a-5p | 6 | -3.13 | 3.38 E-06 |
| miR-100-5p | 8 | -3.07 | 3.76 E-06 |
| miR-125b-5p | 30 | -2.78 | 1.19 E-04 |
| (miR-99b-5p) | (3) | (-1.84) | (1.51 E-08) |
| (miR-125a-5p) | (64) | (-1.16) | (2.85 E-03) |
|  | | | |
| **miRNA** | **Yolk sac tumor (YST)** | | |
|  | Downregulated rank (n=112) | Log_2_ FC | Adjusted *p*-value |
| miR-99a-5p | 5 | -3.51 | 3.94 E-07 |
| miR-100-5p | 2 | -3.7 | 1.26 E-07 |
| miR-125b-5p | 6 | -3.89 | 7.35 E-07 |
| (miR-99b-5p) | (42) | (-1.36) | (1.15 E-04) |
| (miR-125a-5p) | (36) | (-1.66) | (5.55 E-05) |
|  | | | |
| **miRNA** | **Embryonal carcinoma (EC)** | | |
|  | Downregulated rank (n=96) | Log_2_ FC | Adjusted *p*-value |
| miR-99a-5p | 20 | -3.77 | 2.85 E-04 |
| miR-100-5p | 40 | -3.39 | 7.17 E-04 |
| miR-125b-5p | 53 | -3.68 | 1.26 E-03 |
| (miR-99b-5p) | (17) | (-2.19) | (1.73 E-04) |
| (miR-125a-5p) | (2) | (-2.92) | (3.49 E-05) |
|  | | | |
| **miRNA** | **Malignant GCT cell lines** | | |
|  | Downregulated rank (n=52) | Log_2_ FC | Adjusted *p*-value |
| miR-100-5p | 9 | -4.07 | 4.06 E-07 |
| miR-99a-5p | 14 | -3.33 | 1.92 E-05 |
| miR-125b-5p | 6 | -5.02 | 9.98 E-08 |
| (miR-99b-5p) | (-) | (-) | (-) |
| (miR-125a-5p) | (36) | (-1.49) | (2.00 E-03) |

**Supplementary-Table-S7. Downregulation of miR-99a-5p, miR-100-5p, and miR-125b-5p in malignant germ cell tumors (GCTs), GCT subtypes, and cell lines** **relative to non-malignant (gonadal and teratoma) control samples; derived from global microRNA (miRNA) microarray expression data.** Data also shown in parentheses for miR-99b-5p (which shares the 2-7nt seed region with miR-99b-5p/miR-100-5p) and miR-125a-5p (which shares the 2-7nt seed region with miR-125-5p). MiR-99a-5p/miR-100-5p and miR-125b-5p derive from both chromosome 11q24.1 and chromosome 21q21.1; miR-99b-5p and miR-125a-5p derive from chromosome 19q13.41. Data derived from (1).

**Supplementary-Table-S8**

| 1. **Downregulated genes and enrichment for respective miRNA SCR (denominator 1,400 genes)** | | | | |
| --- | --- | --- | --- | --- |
| *miRNA* | *TCam2 d2* | *%* | *1411H d2* | *%* |
| miR-99a-5p/miR-100-5p | 197 | 14.1 | 179 | 12.8 |
| miR-125b-5p | 793 | 56.6 | 830 | 59.3 |
| *miRNA* | *TCam2 d7* | *%* | *1411H d7* | *%* |
| miR-99a-5p/miR-100-5p | 164 | 11.7 | 167 | 11.9 |
| miR-125b-5p | 776 | 55.4 | 805 | 57.5 |

| 1. **Background miRNA SCR occurrence across whole geneset (denominator used 8,706 genes)** | | | | |
| --- | --- | --- | --- | --- |
| *miRNA* | *2-7nt SCR 'ACGGGT'* | *%* | *1-8nt SCR 'TACGGGTA'* | *%* |
| miR-99a-5p/miR-100-5p | 484 | 5.6 | 16 | 0.2 |
| *miRNA* | *2-7nt SCR 'TCAGGG'* | *%* | *1-8nt SCR 'CTCAGGGA'* | *%* |
| miR-125b-5p | 2,768 | 31.8 | 392 | 4.5 |

**Supplementary-Table-S8. Enrichment for seed complementary regions (SCRs) corresponding to the seed regions of miR-99a-5p/miR-100-5p and miR-125b-5p in 3’ untranslated regions (3'UTRs) of messenger RNA (mRNA) data.** A) Number of genes detected for TCam2 and 1411H cell lines, targeted individually by either the microRNAs (miRNAs) miR-99a-5p/100-5p or miR-125b-5p at day 2 (d2) and day 7 (d7) timepoints. The percentages are referenced to the total number of downregulated genes derived from *Sylamer* analysis (total 1,400 genes). B) Background occurrence across the whole geneset of miR-99a-5p/100-5p and miR-125b-5p SCRs referenced to the total number of unique genes with known 3’UTR information (8,706 genes, using TCam2 d2 denominator). Key: nt = nucleotide.

**Supplementary-Table-S9**

| **GO/Reactome term** | **Description** | **Gene Count** | **%** | **Log_10_(p)** | **Log_10_(q)** |
| --- | --- | --- | --- | --- | --- |
| R-HSA-8953854 | Metabolism of RNA | 58 | 6.98 | -12.60 | -8.25 |
| R-HSA-1640170 | Cell Cycle | 54 | 6.50 | -11.16 | -7.11 |
| GO:0000278 | Mitotic cell cycle | 49 | 5.90 | -10.62 | -6.75 |
| GO:1901137 | Carbohydrate derivative biosynthetic process | 46 | 5.54 | -9.92 | -6.17 |
| GO:0034660 | ncRNA metabolic process | 43 | 5.17 | -9.50 | -5.85 |
| GO:0045785 | Positive regulation of cell adhesion | 40 | 4.81 | -9.16 | -5.66 |
| GO:1990778 | Protein localization to cell periphery | 27 | 3.25 | -9.06 | -5.62 |
| GO:0010564 | Regulation of cell cycle process | 51 | 6.14 | -8.56 | -5.26 |
| GO:0030163 | Protein catabolic process | 48 | 5.78 | -7.98 | -4.89 |
| GO:0051336 | Regulation of hydrolase activity | 51 | 6.14 | -7.98 | -4.89 |
| GO:0051301 | Cell division | 39 | 4.69 | -7.89 | -4.82 |
| R-HSA-9716542 | Signaling by Rho GTPases, Miro GTPases, and RHOBTB3 | 48 | 5.78 | -7.70 | -4.67 |
| GO:0030162 | Regulation of proteolysis | 43 | 5.17 | -7.51 | -4.53 |
| GO:0061024 | Membrane organization | 49 | 5.90 | -7.43 | -4.46 |
| GO:0097190 | Apoptotic signaling pathway | 28 | 3.37 | -7.34 | -4.40 |
| GO:0007005 | Mitochondrion organization | 34 | 4.09 | -6.85 | -4.00 |
| GO:0030097 | Hemopoiesis | 44 | 5.29 | -6.83 | -3.99 |
| GO:0051640 | Organelle localization | 38 | 4.57 | -6.79 | -3.97 |
| GO:0007059 | Chromosome segregation | 28 | 3.37 | -6.75 | -3.94 |
| GO:0045936 | Negative regulation of phosphate metabolic process | 30 | 3.61 | -6.66 | -3.88 |

**Supplementary-Table-S9. The top-20 deregulated Metascape pathways in TCam2 cells at day 2 (d2) after combination miR-100-5p/miR-125b-5p replenishment.** The table lists the 20 most deregulated pathways for messenger RNA (mRNA) targets of either miR-100-5p or miR-125b-5p at d2 after treatment, ranked by *p*-value [log_10_(p)], with gene count and percentage (%) gene count per pathway also listed. Log_10_(q) is the multi-test adjusted *p*-value in log base 10.

**Supplementary-Table-S10**

| **GO/Reactome term** | **Description** | **Gene Count** | **%** | **Log_10_(p)** | **Log_10_(q)** |
| --- | --- | --- | --- | --- | --- |
| R-HSA-199991 | Membrane Trafficking | 54 | 6.34 | -12.19 | -7.85 |
| GO:0016310 | Phosphorylation | 54 | 6.34 | -10.03 | -6.17 |
| GO:0030163 | Protein catabolic process | 52 | 6.10 | -9.43 | -5.68 |
| R-HSA-9012999 | RHO GTPase cycle | 38 | 4.46 | -8.68 | -5.16 |
| GO:0016050 | Vesicle organization | 32 | 3.76 | -8.61 | -5.16 |
| GO:1903047 | Mitotic cell cycle process | 41 | 4.81 | -8.36 | -5.06 |
| GO:0031344 | Regulation of cell projection organization | 47 | 5.52 | -7.97 | -4.70 |
| R-HSA-71387 | Metabolism of carbohydrates | 28 | 3.29 | -7.54 | -4.42 |
| GO:1901137 | Carbohydrate derivative biosynthetic process | 41 | 4.81 | -7.19 | -4.12 |
| M72 | Pathway Interaction Database (PID) Nectin pathway | 9 | 1.06 | -7.05 | -4.01 |
| GO:0006886 | Intracellular protein transport | 46 | 5.40 | -6.94 | -3.95 |
| R-HSA-9006934 | Signaling by Receptor Tyrosine Kinases | 38 | 4.46 | -6.89 | -3.93 |
| GO:0051336 | Regulation of hydrolase activity | 49 | 5.75 | -6.83 | -3.88 |
| hsa04142 | Lysosome | 17 | 2.00 | -6.72 | -3.79 |
| GO:0031329 | Regulation of cellular catabolic process | 46 | 5.40 | -6.55 | -3.64 |
| GO:0048193 | Golgi vesicle transport | 25 | 2.93 | -6.36 | -3.50 |
| GO:0034330 | Cell junction organization | 36 | 4.23 | -6.03 | -3.21 |
| GO:0007169 | Transmembrane receptor protein tyrosine kinase signaling pathway | 31 | 3.64 | -5.99 | -3.19 |
| GO:0016236 | Macroautophagy | 20 | 2.35 | -5.94 | -3.17 |
| GO:0016197 | Endosomal transport | 22 | 2.58 | -5.92 | -3.17 |

**Supplementary-Table-S10. The top-20 deregulated Metascape pathways in 1411H cells at day 2 (d2) after combination miR-100-5p/miR-125b-5p replenishment**. The table lists the 20 most deregulated pathways for messenger RNA (mRNA) targets of either miR-100-5p or miR-125b-5p at d2 after treatment, ranked by *p*-value [log_10_(p)], with gene count and percentage (%) gene count per pathway also listed. Log_10_(q) is the multi-test adjusted *p*-value in log base 10.

**Supplementary-Table-S11**

| **GO/Reactome term** | **Description** | **Gene Count** | **%** | **Log_10_(p)** | **Log_10_(q)** |
| --- | --- | --- | --- | --- | --- |
| R-HSA-382551 | Transport of small molecules | 56 | 7.01 | -11.96 | -7.61 |
| GO:0000278 | Mitotic cell cycle | 49 | 6.13 | -11.22 | -7.32 |
| R-HSA-199991 | Membrane Trafficking | 50 | 6.26 | -11.12 | -7.32 |
| GO:0008610 | Lipid biosynthetic process | 48 | 6.01 | -11.07 | -7.32 |
| R-HSA-1640170 | Cell Cycle | 51 | 6.38 | -10.28 | -6.64 |
| GO:0051640 | Organelle localization | 43 | 5.38 | -9.72 | -6.22 |
| GO:0010256 | Endomembrane system organization | 43 | 5.38 | -9.40 | -5.96 |
| R-HSA-9716542 | Signaling by Rho GTPases, Miro GTPases, and RHOBTB3 | 50 | 6.26 | -9.14 | -5.92 |
| WP5087 | Pleural mesothelioma | 37 | 4.63 | -9.14 | -5.92 |
| GO:0080135 | Regulation of cellular response to stress | 47 | 5.88 | -9.12 | -5.92 |
| GO:0001701 | *In utero* embryonic development | 34 | 4.26 | -8.97 | -5.81 |
| GO:0051336 | Regulation of hydrolase activity | 51 | 6.38 | -8.54 | -5.45 |
| hsa05010 | Alzheimer disease | 33 | 4.13 | -8.45 | -5.39 |
| GO:0010564 | Regulation of cell cycle process | 49 | 6.13 | -8.24 | -5.19 |
| GO:1901137 | Carbohydrate derivative biosynthetic process | 41 | 5.13 | -7.95 | -4.95 |
| R-HSA-1280215 | Cytokine Signaling in Immune system | 47 | 5.88 | -7.61 | -4.65 |
| GO:0051056 | Regulation of small GTPase mediated signal transduction | 27 | 3.38 | -7.60 | -4.65 |
| GO:0005975 | Carbohydrate metabolic process | 34 | 4.26 | -7.51 | -4.60 |
| R-HSA-1280218 | Adaptive Immune System | 48 | 6.01 | -7.38 | -4.48 |
| GO:0007167 | Enzyme-linked receptor protein signaling pathway | 41 | 5.13 | -7.13 | -4.26 |

**Supplementary-Table-S11. The top-20 deregulated Metascape pathways in TCam2 cells at day 7 (d7) after combination miR-100-5p/miR-125b-5p replenishment.** The table lists the 20 most deregulated pathways for messenger RNA (mRNA) targets of either miR-100-5p or miR-125b-5p at d7 after treatment, ranked by *p*-value [log_10_(p)], with gene count and percentage (%) gene count per pathway also listed. Log_10_(q) is the multi-test adjusted *p*-value in log base 10.

**Supplementary-Table-S12**

| **GO/Reactome term** | **Description** | **Gene Count** | **%** | **Log_10_(p)** | **Log_10_(q)** |
| --- | --- | --- | --- | --- | --- |
| GO:0006974 | DNA damage response | 62 | 7.46 | -13.70 | -9.35 |
| GO:0097190 | Apoptotic signaling pathway | 34 | 4.09 | -11.04 | -7.17 |
| GO:0009725 | Response to hormone | 54 | 6.50 | -9.33 | -5.58 |
| WP5087 | Pleural mesothelioma | 38 | 4.57 | -9.22 | -5.58 |
| R-HSA-8953854 | Metabolism of RNA | 51 | 6.14 | -9.15 | -5.58 |
| GO:0031329 | Regulation of cellular catabolic process | 51 | 6.14 | -9.02 | -5.55 |
| GO:0051603 | Proteolysis involved in protein catabolic process | 45 | 5.42 | -8.99 | -5.55 |
| GO:0006325 | Chromatin organization | 53 | 6.38 | -8.94 | -5.55 |
| R-HSA-1640170 | Cell Cycle | 49 | 5.90 | -8.75 | -5.44 |
| GO:0030162 | Regulation of proteolysis | 44 | 5.29 | -7.97 | -4.77 |
| R-HSA-109581 | Apoptosis | 21 | 2.53 | -7.56 | -4.48 |
| M48 | Pathway Interaction Database (PID) MET PATHWAY | 14 | 1.68 | -7.54 | -4.48 |
| GO:0044092 | Negative regulation of molecular function | 50 | 6.02 | -7.32 | -4.33 |
| GO:0032774 | RNA biosynthetic process | 38 | 4.57 | -7.25 | -4.29 |
| R-HSA-3700989 | Transcriptional Regulation by TP53 | 30 | 3.61 | -7.02 | -4.12 |
| GO:0043549 | Regulation of kinase activity | 40 | 4.81 | -6.91 | -4.06 |
| GO:0051052 | Regulation of DNA metabolic process | 37 | 4.45 | -6.65 | -3.87 |
| GO:0031344 | Regulation of cell projection organization | 43 | 5.17 | -6.57 | -3.82 |
| R-HSA-2262752 | Cellular responses to stress | 48 | 5.78 | -6.56 | -3.82 |
| GO:0016310 | Phosphorylation | 45 | 5.42 | -6.46 | -3.76 |

**Supplementary-Table-S12. The top-20 deregulated Metascape pathways in 1411H cells at day 7 (d7) after combination miR-100-5p/miR-125b-5p replenishment**. The table lists the 20 most deregulated pathways for messenger RNA (mRNA) targets of either miR-100-5p or miR-125b-5p at d7 after treatment, ranked by *p*-value [log_10_(p)], with gene count and percentage (%) gene count per pathway also listed. Log_10_(q) is the multi-test adjusted *p*-value in log base 10.

**Legends to Supplementary Tables S13-S16 (see separate excel files)**

**Supplementary-Table-S13. The list of 832 downregulated genes following combination 16.7nM miR-100-5p/miR-125b-5p replenishment of TCam2 (seminoma) malignant germ cell tumor (GCT) cells at day 2 (d2) following transfection which were either miR-100-5p targets (n=197), miR-125b-5p targets (n=793), or both (n=158).** Whether these genes are miR-100-5p targets, miR-125b-5p targets, or both are listed as a binary outcome (1= target; 0 = not a target). Similarly, for these 832 genes used in the Metascape analysis, whether individual genes are involved in the top-20 Metascape pathways are listed as a binary outcome (1 = involved; 0 = not involved).

**Supplementary-Table-S14. The list of 852 downregulated genes following combination 16.7nM miR-100-5p/miR-125b-5p replenishment of 1411H (yolk sac tumor) malignant germ cell tumor (GCT) cells at day 2 (d2) following transfection which were either miR-100-5p targets (n=179), miR-125b-5p targets (n=830), or both (n=157).** Whether these genes are miR-100-5p targets, miR-125b-5p targets, or both are listed as a binary outcome (1= target; 0 = not a target). Similarly, for these 852 genes used in the Metascape analysis, whether individual genes are involved in the top-20 Metascape pathways are listed as a binary outcome (1 = involved; 0 = not involved).

**Supplementary-Table-S15. The list of 800 downregulated genes following combination 16.7nM miR-100-5p/miR-125b-5p replenishment of TCam2 (seminoma) malignant germ cell tumor (GCT) cells at day 7 (d7) following transfection which were either miR-100-5p targets (n=164), miR-125b-5p targets (n=776), or both (n=140).** Whether these genes are miR-100-5p targets, miR-125b-5p targets, or both are listed as a binary outcome (1= target; 0 = not a target). Similarly, for these 800 genes used in the Metascape analysis, whether individual genes are involved in the top-20 Metascape pathways are listed as a binary outcome (1 = involved; 0 = not involved).

**Supplementary-Table-S16. The list of 831 downregulated genes following combination 16.7nM miR-100-5p/miR-125b-5p replenishment of 1411H (yolk sac tumor) malignant germ cell tumor (GCT) cells at day 7 (d7) following transfection which were either miR-100-5p targets (n=167), miR-125b-5p targets (n=805), or both (n=141).** Whether these genes are miR-100-5p targets, miR-125b-5p targets, or both are listed as a binary outcome (1= target; 0 = not a target). Similarly, for these 831 genes used in the Metascape analysis, whether individual genes are involved in the top-20 Metascape pathways are listed as a binary outcome (1 = involved; 0 = not involved).

**Legends to Supplementary Figures S1-S11**

**Supplementary-Figure-S1. Confirmatory quantitative RT-PCR (qRT-PCR) data showing relative miR-99a-5p/miR-100-5p and miR-125b-5p expression in malignant germ cell tumor (GCT) clinical samples (n=24) and cell lines (n=7) compared with controls (n=2).** A) Relative miR-99a-5p/miR-100-5p expression levels and B) Relative miR-125b-5p expression levels. These data confirm downregulation of both of these microRNAs (miRNAs) in malignant GCT samples and cell lines, consistent with previous global miRNA microarray data (1). C) Linear regression analysis confirming a highly significant positive correlation of miR-125b-5p *versus* miR-99a-5p/miR-100-5p expression levels. Expression levels are relative to pooled gonadal control (i.e., the mean expression of the ovary and testis samples; n=2) and represented as log_10_ transformed values. Color-coding for all panels is as per the key shown in C).

**Supplementary-Figure-S2. Genomic loci and expression levels of genes of interest on chromosomes 11 and 21 in malignant germ cell tumors (GCTs).** A) Genomic overview of the microRNA (miRNA) clusters of interest. Chromosome 11 (chr11) locus containing miR-100/miR-125b-1, the protein-coding gene *BLID*, and the long interspersed non-coding RNA (lincRNA) *MIR100HG* (left). Chromosome 21 (chr21) locus containing miR-99a/miR-125b-2 and the lincRNA *MIR99AHG* (also termed *LINC00478*) (right). B) Expression of primary microRNAs (pri-miRNAs) of interest in representative malignant GCT cell lines. C) Expression of the lincRNAs of interest and the *BLID* gene in representative malignant GCT cell lines. In both B) and C), gray and black bars represent chr11 (where miR-100/miR-125b-1 are located) and chr21 (miR-99a/miR-125b-2) contributions, respectively, and data are presented as mean ± standard-error-of-the-mean (SEM) of n=3 biological replicates.

**Supplementary-Figure-S3. Genomic copy number data** **across the regions of interest** **on chromosomes 11 and 21 in malignant germ cell tumors (GCTs).** No evidence of consistent overall genomic copy number loss for chromosome 11 (chr11; left) or chromosome 21 (chr21; right) to account for the observed downregulation of miR-99a-5p/miR-100-5p and miR-125b-5p across the four malignant GCT cell lines interrogated, referenced to pooled gonadal control (i.e., the mean copy number of the ovary and testis samples, arbitrarily assigned a copy number of 1.0). Seven and eight sites across the regions of interest were assessed for chr11 and chr21, respectively, and normalized to four housekeeping genes. Graphs show the median expression across the sites assessed as boxplots, with the maximum and minimum ranges displayed. Note that for chr11, genomic copy number for TCam2 and 1411H cell lines was not lower than the range observed in normal testis.

**Supplementary-Figure-S4. Overall evidence that hypermethylation at chromosome 11 and 21 microRNA (miRNA) loci contributes to miR-99a-5p/miR-100-5p, miR-125b-5p, and related long interspersed non-coding RNA (lincRNA) and protein-coding gene downregulation in malignant germ cell tumors (GCTs) and cell lines.** A variety of complementary approaches were utilised for this work. Expression of A) miR-100-5p (left) and miR-125b-5p (right) and B) *MIR100HG* [on chromosome 11 (chr11); left] *BLID* (chr11; middle), and *MIR99AHG* [on chromosome 21 (chr21); also termed *LINC00478*; right] in malignant GCT cells on day 3 (d3) and day 4 (d4) following 5µM 5-azacytidine treatment. In both A) and B), expression is normalized to the respective day-matched untreated control, and data presented as mean ± standard-error-of-the-mean (SEM) of n=3 biological replicates. Statistical significance for malignant GCT cells compared with relevant untreated cells in both A) and B): p*<0.05, **p<0.01, ***p<0.005, ****p<0.001. Note that due to some variability in results from repeat 5-azacytidine experiments, despite overall increases in levels across the genes tested, only a proportion were statistically significant. C) Chromosome location of miR-100 and miR-125b-1 on the reverse strand of chr11 (upper schematic) and of miR-99a and miR-125b-2 on the forward strand of chr21 (lower schematic) using Ensembl genomic coordinates [version GRCh38.p14]. D) Global published methylation data from clinical samples and cell lines using the NCBI Genome Data Viewer, showing hypermethylation in malignant GCT tissues and cell lines compared with teratoma controls. Beta and derived median (M) values from specific probes were compared across sample types. M values can be negative as they are calculated using M value = log_2_(beta/1-beta). An M value close to 0 indicates a similar intensity between methylated and unmethylated probes, i.e., ~50% methylated. Thus, teratomas, with an M value of -4.9 are relatively hypomethylated compared with the more methylated seminoma and NSGCT tissue samples and cell lines.

**Supplementary-Figure-S5. Lack of phenotypic effects of combination miR-100-5p and miR-125b-5p replenishment in 2102Ep (embryonal carcinoma) malignant GCT cells and potential explanation through enhanced excretion in extracellular vesicles (EVs).** A) Growth curves for 2102Ep cells after transfection with total 16.7nM (left), 33.3nM (middle) and 66.7nM (right) equimolar concentration of combination miR-100-5p and miR-125b-5p mimics, compared with the relevant mimic-negative-control (MNC)-treated cells (all non-significant). B) Quantification of the replenished microRNAs (miRNAs) miR-100-5p and miR-125b-5p in recipient TCam2 (left), 1411H (middle), and 2102Ep (right) cells treated with 16.7nM combination mimic replenishment after days (d) 2, 4 and 7 (d2, d4, and d7) of treatment. Key: blue bars = miR-100-5p levels; green bars = miR-125b-5p levels. C) In further 16.7nM combination mimic replenishment experiments, the replenished miRNAs miR-100-5p and miR-125b-5p were quantified in recipient 1411H (left upper panel) and 2102Ep (right upper panel) cells after d1 and d2 of treatment. Levels were also quantified in extracellular vesicles (EVs) extracted from the cell media for 1411H (left lower panel) and 2102Ep (right lower panel) at identical timepoints. EV miR-100-5p and miR-125b-5p levels relative to the intracellular levels on d1 are listed above the relevant bars on the graphs. Key: blue bars = miR-100-5p levels; green bars = miR-125b-5p levels.

**Supplementary-Figure-S6. Using the change-point detection algorithm to determine the optimal peaks in the *Sylamer* landscape plots at day 2 (d2) following 16.7nM combination miR-100-5p/miR-125b-5p replenishment.** Following generation of *Sylamer* landscape plots of single summed significance scores (SSSS), the change-point detection algorithm was employed to identify the most appropriate enrichment peak for selecting gene lists for further analyses at d2 following combination miR-100-5p/miR-125b-5p replenishment at 16.7nM. This algorithm computed a change-point delta value (CPDV) for each bin of 200 genes (*x*-axis), based on the difference between its -log_10_(*p*-value) and the minimum -log_10_(*p*-value) across the next five bins (progressing from left to right), as described (2). Here, CPDV curves were averaged for the two seed complementary regions (SCRs) of interest, corresponding to the two 2-7 nucleotide (nt) microRNA seeds, and plotted, and the bin with the maximum average CPDV selected. A) TCam2 cell line; bin 7 selected (vertical line; 1,400 genes); B) 1411H cell line; bin 7 selected (vertical line; 1,400 genes).

**Supplementary-Figure-S7. *Sylamer* assessment for persistent seed complementary region (SCR) enrichment in downregulated genes at day 7 (d7) following combination miR-100-5p/miR-125b-5p replenishment at 16.7nM.** *Sylamer* plots showing the single summed significance score (SSSS) of the seed complementary regions (SCRs) corresponding to miR-99a-5p/miR-100-5p (red line) and miR-125b-5p (blue line) in the ranked genelists from TCam2 (left), 1411H (centre), and 2102Ep (right) cells at d7 following treatment with combination miR-100-5p and miR-125b-5p mimic replenishment, compared with mimic-negative-control (MNC)-treated cells at 16.7nM. Log_10_-transformed *p*-values for each SCR word are on the *y*-axis, against the ranked gene list (from downregulated on the left to upregulated on the right) on the *x*-axis. A positive *y*-axis deflection on the left-hand side of the plot signifies SCR enrichment in downregulated genes. *Sylamer* analysis demonstrated that enrichment for the SCRs corresponding to miR-99a-5p/miR-100-5p and miR-125b-5p in downregulated genes persisted to d7 in both TCam2 and 1411H cell lines [i.e., above the red dotted horizontal -log_10_(*p*-value) threshold of 4], the timepoint when significant growth inhibition was seen. However, as expected, such genotypic changes were not present in 2102Ep EC cells at d7, consistent with the lack of growth phenotype observed at this timepoint.

**Supplementary-Figure-S8: Schematic showing the number of downregulated miR-99a-5p/miR-100-5p or miR-125b-5p mRNA target genes in malignant germ cell tumor (GCT) cell lines following** **combination miR-100-5p/miR-125b-5p replenishment at 16.7nM.** Venn diagrams showing the number of downregulated genes containing the seed complementary regions (SCRs) for either miR-99a-5p/miR-100-5p or miR-125b-5p in TCam2 (seminoma) cells (left) and 1411H (yolk sac tumor) cells (right). A) Data for day 2 (d2) post-replenishment; B) Data for day 7 (d7) post-replenishment. The total number of target genes for each cell line at each timepoint is listed. The total number of downregulated genes for each comparison (total denominator) is 1,400, derived from *Sylamer* change-point delta value (CPDV) analysis.

**Supplementary-Figure-S9. Negative correlation between miR-100-5p and miR-125b-5p levels versus *FGFR3*, *ARID3B*, and *E2F7* messenger RNA (mRNA) levels in malignant germ cell tumor (GCT) tissue samples.** Co-expression analysis of miR-100-5p levels (left) and miR-125b-5p levels (right) (*x*-axes) with A) *FGFR3*, B) *ARID3B*, and C) *E2F7* mRNA levels (*y*-axes) in malignant GCT tissue samples (n=156). Data obtained from the independent ENCORI Pan-Cancer Analysis Platform.

**Supplementary-Figure-S10. Metascape pathway analysis in malignant germ cell tumour (GCT) cells at day 7 (d7) following combination miR-100-5p and miR-125b-5p replenishment at 16.7nM.** Bar plots and associated network analyses for A) TCam2 (d7; 800 genes), and B) 1411H (d7; 831 genes) showing the top-20 most significant functional pathways for the downregulated seed complementary region (SCR)-containing messenger RNA (mRNA) targets of miR-100-5p or miR-125b-5p following combination microRNA replenishment.

**Supplementary-Figure-S11. Metascape pathway analysis on the 734 unique upregulated miR-100-5p or miR-125b-5p messenger RNA (mRNA) targets from clinical malignant germ cell tumor (GCT) tissue samples.** A) Venn diagram showing miR-100-5p targets (n=114), miR-125b-5p targets (n=683), and overlap of 63 targets, leaving a total of 734 unique targets of either miR-100-5p or miR-125b-5p. B) Metascape bar plot showing the top-20 most significant functional pathways for the 734 upregulated seed complementary region (SCR)-containing messenger RNA (mRNA) targets of miR-100-5p or miR-125b-5p. Malignant GCT tissue data derived from (1, 3).

**Supplementary References**

1. Palmer RD, Murray MJ, Saini HK, van Dongen S, Abreu-Goodger C, Muralidhar B, et al. Malignant germ cell tumors display common microRNA profiles resulting in global changes in expression of messenger RNA targets. Cancer Res. 2010;70(7):2911-23.

2. Bailey S, Ferraresso M, Alonso-Crisostomo L, Ward D, Smith S, Nicholson JC, et al. Targeting oncogenic microRNAs from the miR-371~373 and miR-302/367 clusters in malignant germ cell tumours causes growth inhibition through cell cycle disruption. Br J Cancer. 2023.

3. Murray MJ, Saini HK, Siegler CA, Hanning JE, Barker EM, van Dongen S, et al. LIN28 Expression in malignant germ cell tumors downregulates let-7 and increases oncogene levels. Cancer Res. 2013;73(15):4872-84.
